# Tape-Casting BaTiO_3_/P(VDF-TrFE) Piezoelectric Composite Films with Optimized Ferroelectric–Dielectric Performances for Wearable Human Motion Monitoring

**DOI:** 10.3390/polym18202518

**Published:** 2026-10-14

**Authors:** Mao Cai, Zhineng Tan, Shengwen Liu, Zhenghang Chen, Xi Yuan, Dou Zhang

**Affiliations:** 1 College of Chemistry and Chemical Engineering, Central South University, Changsha 410083, China; 242312172@csu.edu.cn (M.C.);; 2 State Key Laboratory of Powder Metallurgy, Central South University, Changsha 410083, China

**Keywords:** piezoelectric sensors, motion detection, cyclic stability, BaTiO_3_ nanoparticles, ferroelectricity

## Abstract

Piezoelectric sensors have been widely adopted in passive sensing applications owing to their self-powered nature, and they play a significant role in next-generation wearable electronics. Among existing piezoelectric materials, flexible P(VDF-TrFE)-based composites have emerged as promising candidates for fabricating high-performance sensors. However, pure P(VDF-TrFE) suffers from poor piezoelectric performance, which makes it difficult to achieve accurate motion detection because of its weak piezoelectric output and low signal-to-noise ratio. In this study, BaTiO_3_ (BT), which exhibits a significantly higher d_33_ than the polymer matrix, was introduced into P(VDF-TrFE) to fabricate piezoelectric films via the tape casting method. The piezoelectric, ferroelectric, and dielectric properties of the films were systematically investigated at various BT loading concentrations. At a loading concentration of 15%, the absolute d_33_ value reached 27.6 pC/N, representing a 50.8% increase compared with 18.3 pC/N for the unloaded film, and the β-phase content reached 86.1%. Under an external force of 50 N, the sensor delivered a piezoelectric output of 2.30 V, demonstrating excellent output and waveform characteristics for motion monitoring. Under five discrete levels of applied force, the sensor generated distinguishable piezoelectric outputs with excellent reproducibility. Based on the linear relationship between the output voltage and the external force, the sensor sensitivity was determined to be 0.0515 V/N. During the cyclic stability test, the piezoelectric output of the as-fabricated sensor exhibited a slight degradation of approximately 3% after 10,000 consecutive loading cycles, demonstrating satisfactory long-term operational stability. Furthermore, ferroelectric and dielectric analysis revealed that the maximum polarization and remanent polarization increased significantly after BT loading, while the dielectric properties exhibited a monotonic increase in permittivity while maintaining low dielectric loss. In summary, this sensor holds great potential for monitoring joint movement during physical activity.

## 1. Introduction

The growing emphasis on athletic performance and health management has spurred considerable interest in continuous physical activity monitoring [1,2,3]. Meanwhile, the aging population has further driven the demand for real-time health monitoring, thereby promoting the advancement of sensor technology [4,5,6,7]. Sensors are commonly fabricated from materials that exhibit piezoelectric, piezoresistive [8,9], or triboelectric [10,11] properties. Among these, owing to their self-powered nature, excellent integration ability, and superior dynamic force response [12,13,14], piezoelectric sensors are widely used in fields such as robotic electronic skin and human-computer interaction [15,16,17].

Piezoelectric sensors are mainly divided into three categories: piezoelectric polymers, piezoelectric ceramics, and piezoelectric single crystals. However, piezoelectric ceramics are not suitable for fabricating flexible wearable devices because of their inherent brittleness. In contrast to piezoelectric ceramics, piezoelectric polymers offer excellent flexibility and tensile properties, rendering them more promising for the fabrication of flexible wearable devices. Nevertheless, P(VDF-TrFE) poses significant challenges in the fabrication of high-performance sensing devices due to its low piezoelectric coefficient and inferior cyclic stability [18,19]. Therefore, developing piezoelectric sensors that combine long-term durability and superior sensing capability remains a critical pursuit.

In recent years, to improve the sensing performance of P(VDF-TrFE)-based flexible piezoelectric sensors, researchers have incorporated inorganic piezoelectric fillers such as BT [20], PZT [21], and ZnO [22]. Among these fillers, BT has become a widely used material for enhancing the piezoelectric coefficient of P(VDF-TrFE) due to its lead-free nature and excellent biocompatibility. BT nanoparticles are uniformly dispersed in the P(VDF-TrFE) matrix, and corona poling is then applied to increase the β-phase content [23] in the polymer, ultimately achieving the objective of enhancing the piezoelectric coefficients of the composite films. Fabrication protocols profoundly govern the resulting properties, even for identical material systems. Hoejin Kim et al. utilized 3D printing technology to prepare films with excellent dispersion by combining BaTiO_3_ with a PVDF matrix through two steps: filament extrusion and 3D printing [24]. They further enhanced the piezoelectric response via thermal polarization, achieving a threefold improvement in piezoelectric performance compared to the solution casting method. B. S. Athira et al. loaded PVDF with BaTiO_3_ and used electrospinning to prepare films with a β-phase content as high as 91% [25]. The resulting nanogenerator delivered an open-circuit voltage of 50 V and a short-circuit current density of approximately 0.312 mA/m^2^. Furthermore, the piezoelectric nanogenerators produced using this spinning technique exhibited a power density approximately 10 times higher than those made from unloaded materials. In addition, the particle size and dimensionality of piezoelectric ceramics also influence the material’s final performance. Smaller piezoelectric ceramic particles tend to disperse more uniformly in the solution, thereby improving the piezoelectric performance of the resulting composite, provided that agglomeration is avoided [26]. Filler dimensionality is primarily classified into 0D [27] (piezoelectric particles), 1D [28] (piezoelectric nanowires), and 2D [29] (piezoelectric nanosheets), among others. Compared with 0D fillers, 1D and 2D fillers can enhance the dielectric constant and polarization strength of nanocomposite films owing to their larger dipole moments; however, their orientation and dispersion characteristics may lead to a reduction in the breakdown field strength. Furthermore, they are more prone to stress concentration, which can result in performance degradation or even material fracture [30,31]. For example, Kyungwho Choi et al. compared randomly oriented ZnO nanowires that had not undergone polarization treatment with aligned ZnO nanowires that had undergone polarization treatment [32]. They found that the dielectric constant increased by 400% in the aligned state, which clearly demonstrates that orientation plays a crucial role when one-dimensional nanowires are used as fillers. In this context, 0D nanoparticles offer the advantage of isotropic dispersion and reduced stress concentration, making them a pragmatic choice for fabricating homogeneous composite films via tape casting.

In addition, researchers have investigated the effects of post-treatment on the properties of piezoelectric materials [33,34,35]. I.O. Pariy et al. prepared PVDF microcolumns via the imprinting method and then treated the material using hot pressing and high-voltage polarization [36]. Compared to the untreated material, the crystallinity of the treated material increased from 12% to 22.7%. Furthermore, PFM measurements revealed a d_33_ value of 29 pm/V, which was significantly higher than that of the annealed sample (10 pm/V). Maya Sharma et al. systematically investigated the effects of various post-treatments (annealing, polarization, mechanical rolling) and their combinations on the dielectric and ferroelectric properties of PVDF [37]. The results indicated that all three post-treatment methods and their combinations could enhance the material’s ferroelectric and dielectric properties, with mechanical rolling increasing the β-phase content in PVDF to 85%. Maksym Krutko et al. investigated the effects of annealing at four temperatures (80, 100, 120, and 140 °C) on the properties of PVDF-TrFE. The study showed that the β-phase content and crystallinity peaked at 140 °C, and the material exhibited excellent elastic modulus and tensile strength at this temperature [38]. Finally, the modification of the filler material also has a significant impact on material performance [39,40,41,42]. Haowei Lu et al. prepared Tb-modified BCZT particles via freeze-drying and used coaxial electrospinning technology to fabricate 3D-Tb-BCZT/PVDF fibers with a 3D core-shell structure. The PENG output of this fiber reached as high as 48.5 V/3.35 μA, a significant increase compared to the 15.6 V/1.32 μA of the unmodified version [43]. Yuanjie Su et al. used PDA to coat the surface of BaTiO_3_, resulting in better matching of the interfacial modulus between BaTiO_3_ particles and PVDF, which improved piezoelectric performance and led to excellent performance in subsequent applications such as breath detection and facial expression recognition [44]. Shiwen Wang et al. prepared F-BaTiO_3_ by annealing BaTiO_3_ and PVDF powders and then used electrospinning technology to prepare composite nanofibers from a dispersion of F-BaTiO_3_ and PVDF, which were subsequently fabricated into a PENG. The study indicated that at a F-BaTiO_3_ loading level of 5 wt%, the β-phase content reached as high as 91%, and the PENG exhibited a piezoelectric output of up to 1.5 V under an external force of 2 N [45].

In this work, we propose a facile and effective strategy to fabricate flexible piezoelectric films by incorporating BaTiO_3_ (BT) nanoparticles into a P(VDF-TrFE) matrix via the tape-casting method. The effects of BT loading on the microstructural, dielectric, ferroelectric, and piezoelectric properties of the composite films are systematically investigated. To ensure the reliability of the sensing performance, the true piezoelectric response was rigorously decoupled from potential triboelectric interference through comparative polarization studies. Furthermore, the optimized composite films are assembled into flexible sensors to evaluate their sensitivity and long-term cyclic durability under external forces. Ultimately, this study demonstrates the practical viability of the fabricated sensors for continuous human motion monitoring, providing a straightforward yet robust pathway for designing highly reliable piezoelectric sensors for next-generation wearable electronics. The salient contributions of this work can be summarized as follows:

(1) Process innovation: tape-casting for freestanding films with controlled architecture and enhanced durability. Departing from the prevailing electrospinning and solution-casting paradigms, we introduce tape-casting to fabricate freestanding, large-area P(VDF-TrFE)/BaTiO_3_ composite films with precisely controlled thickness and uniform filler dispersion. This dense, homogeneous architecture achieves a competitive piezoelectric coefficient of d_33_ = 27.6 pC/N and a β-phase content of 86.1% without resorting to complex interfacial modification or surface functionalization. Importantly, the tape-cast films exhibit a dense, uniform, and defect-free microstructure, which contributes to excellent mechanical robustness. As demonstrated by the cyclic fatigue test, the optimized composite retains stable piezoelectric output after 10,000 consecutive loading cycles with only 3% degradation, confirming its suitability for long-term wearable applications where repeated mechanical deformation is unavoidable.

(2) Holistic optimization of processing parameters with particular attention to poling protocols for the film architectures. Rather than pursuing single-parameter maximization, we adopt a systematic optimization strategy that balances piezoelectric response, mechanical strength, and processability. Beyond tailored filler loading and post-annealing conditions, we identify and optimize two critical parameters that become increasingly consequential in film systems: (i) poling temperature, which governs ferroelectric domain mobility and enables efficient dipole alignment at moderate electric fields, thereby circumventing dielectric breakdown risks associated with high-voltage poling across a thicker cross-section, and (ii) gold sputtering configuration (electrode geometry and deposition protocol), which ensures uniform electric field distribution throughout the full film thickness and suppresses edge-field concentration that commonly leads to incomplete poling or localized electrical failure in composite materials. Integrated COMSOL Multiphysics® version 5.6 (COMSOL AB, Stockholm, Sweden) finite element analysis further reveals that performance degradation beyond 15 wt% filler loading arises from aggravated electric field concentration and localized stress singularities, establishing a coupled experimental–computational framework that provides mechanistic guidance for the rational design of composite piezoelectric materials.

## 2. Materials and Methods

### 2.1. The Process of Fabricating Piezoelectric Films

All materials and laboratory supplies used in this experiment were of analytical grade, and deionized water was used throughout the experimental process. P(VDF-TrFE) (FC-30) was purchased from ARKEMA, La Défense, France. Barium titanate (200–300 nm, tetragonal phase) was purchased from Macklin, Shanghai, China. N, N-dimethylformamide (DMF, M = 73.1 g/mol) was obtained from Sinopharm Chemical Reagent Co., Ltd. (Shanghai, China). TPU soft films were purchased from YAYUAN Medical Technology Co., Ltd., Wuhan, China. Custom-made copper sheets (15 mm × 15 mm × 0.1 mm) served as electrode materials on both ends.

The process of preparing BT/P(VDF-TrFE) piezoelectric films via tape casting was as follows: First, 7.2 g of DMF was placed on a heating platform and preheated with stirring at 68 °C and 200 rpm to ensure uniform temperature distribution. Then, a total of 0.8 g of BT nanoparticles and P(VDF-TrFE) powder was weighed. The corresponding masses for different BT contents are listed in Table 1. Subsequently, the P(VDF-TrFE) powder was slowly added into the vial to prevent it from wetting and agglomerating on the walls.

After adding the P(VDF-TrFE) powder, the stirring speed was adjusted to 600 rpm, and the mixture was heated and stirred for 4 h. The solution became clear, became viscous, and took on a slight yellow color. BT nanoparticles were then added, and the mixture was heated and stirred for an additional 2 h. Similar to the addition of the P(VDF-TrFE) powder, care was taken to avoid adding the BT nanoparticles onto the inner wall of the vial, as this could compromise the homogeneity of the dispersion. After a total stirring time of 6 h, the heating and stirring were stopped. The stirrer was removed, and the solution was degassed under vacuum to eliminate bubbles that could cause defects in the subsequently prepared films, thereby affecting material properties. Vacuum degassing was performed by placing the vial in a vacuum drying oven, evacuating to −0.1 MPa (gauge pressure), and holding for 5 min. Subsequently, the degassed solution was preheated in a drying oven for 3 min and then uniformly applied onto the preheated glass substrate using a 400 μm thick squeegee to ensure a consistent coating thickness. The coated substrate was immediately transferred to an oven at 75 °C for 1 h to thoroughly remove residual solvent and promote the molecular chain rearrangement and crystallization of P(VDF-TrFE). The entire process was conducted in a clean, dry environment to prevent ambient moisture from interfering with film formation. After drying, P(VDF-TrFE) films with varying BT contents were obtained and cut into 2.0 cm × 2.0 cm squares.

### 2.2. The Process of Making Piezoelectric Sensors

**Figure 1:**
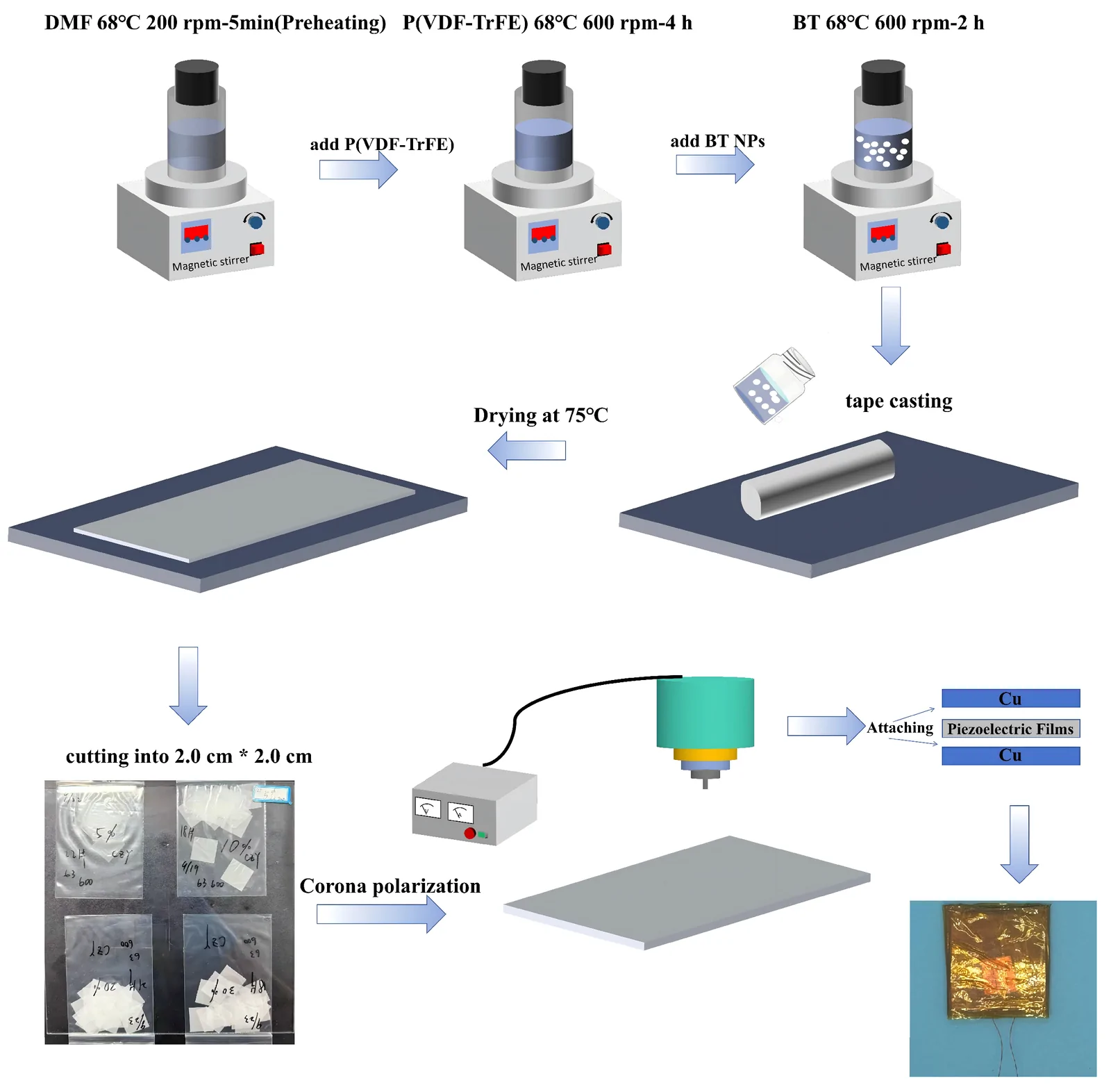
The fabrication process of piezoelectric sensors.

The preparation process of the BT/P(VDF-TrFE)-based piezoelectric sensors is shown in Figure 1 (the detailed photo of the sensor is provided in Figure S1). The prepared piezoelectric films were coated with a single electrode using magnetron sputtering. The corona poling device was set to a temperature of 75 °C and a voltage of 16 kV. A single metal needle was placed 3 cm above the sample surface during corona poling. Corona poling was performed for 40 min. After allowing the prepared films to rest for one day, another electrode was deposited on the opposite side via magnetron sputtering, followed by d_33_ measurement. Two thin copper electrodes were positioned and secured on both sides of the piezoelectric films. Subsequently, TPU films were laminated onto both sides of the electrodes. Through this method, the final piezoelectric sensors were fabricated.

### 2.3. Characterization and Electrical Measurements

#### 2.3.1. Piezoelectric Output and d_33_ Measurement

For piezoelectric characterization, one surface of the as-cast film was first sputter-coated with a gold electrode via magnetron sputtering to ensure adequate electrical conductivity. The film was then subjected to corona poling at 75 °C under a voltage of 16 kV for 40 min with a single metallic needle positioned 3 cm above the sample surface. Following poling, the opposite surface was sputter-coated with another gold electrode to enhance charge collection and transfer during subsequent electrical measurements.

The piezoelectric charge coefficient d_33_ was measured using a quasi-static Berlincourt-type d_33_ meter (ZJ-4AN, Institute of Acoustics, Chinese Academy of Sciences, Beijing, China) at room temperature. During testing, the poled surface was oriented facing upward, and an alternating compressive force of 0.25 N at 110 Hz was applied. For each sample, d_33_ values were measured at five different locations and averaged to ensure statistical reliability. It is noted that the measured d_33_ values exhibited negative polarity across all BT loading concentrations, which is consistent with the polarization orientation established under the present corona poling configuration.

Subsequently, the poled film was assembled into a complete sensor device by laminating thin copper sheets (15 mm × 15 mm × 0.1 mm) onto both sides as top and bottom electrodes, followed by encapsulation with TPU films serving as flexible protective layers. The piezoelectric output performance was evaluated under controlled mechanical excitation using a programmable linear motor (LinMot PS01-23 × 80, NTI AG, Spreitenbach, Switzerland) fitted with an insulating alumina ceramic indenter with a contact area of 5 cm × 5 cm, which fully covered the 1.5 cm × 1.5 cm active area of the sensor to ensure uniform stress distribution. A static preload of 0.5 N was first applied by advancing the indenter into gentle contact with the sensor surface. After the preload had stabilized, the motor speed, acceleration, and displacement amplitude were adjusted so that the peak dynamic force, monitored via the force sensor, reached 10 N. Under these settings, the linear motor delivered a reproducible compressive semi-sinusoidal force waveform at a frequency of 1 Hz, and the resulting piezoelectric output was recorded by a Keithley 6517B Electrometer, Keithley Instruments, Solon, OH, USA (input impedance > 200 TΩ, maximum reading rate of 425 readings/s). It should be noted that the force sensor used in this study provides peak force readings rather than a continuous force–time history; the reported force values therefore correspond to the calibrated peak readings obtained under each motor displacement setting. A periodic compressive impact was applied by controlling the motor stroke, with the impact force modulated by adjusting the displacement amplitude and the contact distance between the motor-driven indenter and the sensor surface. Tests were conducted at a frequency of 2 Hz with calibrated impact forces from 10 to 50 N. Higher-force characterization (10–50 N) was performed in an impact mode at 2 Hz, as the linear motor cannot supply the required thrust under quasi-static preload operation. The output voltage of the flexible piezoelectric sensor was measured by a Keithley 6517B electrometer.

#### 2.3.2. The Dielectric Testing Procedure

For dielectric spectroscopy measurements, the unpoled films were first placed in a shadow mask, and circular gold electrodes were deposited on both surfaces via magnetron sputtering to form a parallel-plate capacitor geometry. The dielectric constant and dielectric loss were then measured using a precision impedance analyzer (E4990A, Keysight, Santa Rosa, CA, USA) over a frequency range of 40 Hz to 10 MHz at room temperature. The capacitance (C) was recorded, and the relative permittivity (ε_r_) was calculated according to the following parallel-plate capacitor formula:

$$\varepsilon_{r}=\frac{C}{\varepsilon_{0}}\cdot \frac{d}{A}$$

where d is the film thickness, A is the electrode area, and ε_0_ is the vacuum permittivity (8.854 × 10^−12^ F/m).

For temperature-dependent dielectric measurements, the electrode area and film thickness were preloaded into the instrument software. The samples were mounted in a temperature-controlled stage, and the dielectric properties were measured from 30 °C to 120 °C at a heating rate of 1 °C/min.

#### 2.3.3. The Ferroelectric Testing Procedure

For ferroelectric characterization, circular gold electrodes (diameter 3 mm) were deposited on both surfaces of the unpoled films (average thickness: 10 μm, variation within ±3%) via magnetron sputtering using a shadow mask. The electrode area and the as-measured film thickness were input into the ferroelectric test system (aixACCT TF Analyzer 2000, aixACCT Systems GmbH, Aachen, Germany.) as geometric correction parameters. P–E hysteresis loops were measured at room temperature. The measurement commenced at an applied electric field of 100 kV/cm; the field amplitude was subsequently raised in increments of 100 kV/cm, with the upper limit determined by the dielectric breakdown strength of each individual sample. The reported P–E data were recorded under a maximum electric field of 2000 kV/cm at a frequency of 1 Hz.

## 3. Results

### 3.1. Microstructural Characterization

#### 3.1.1. XRD and FTIR

**Figure 2:**
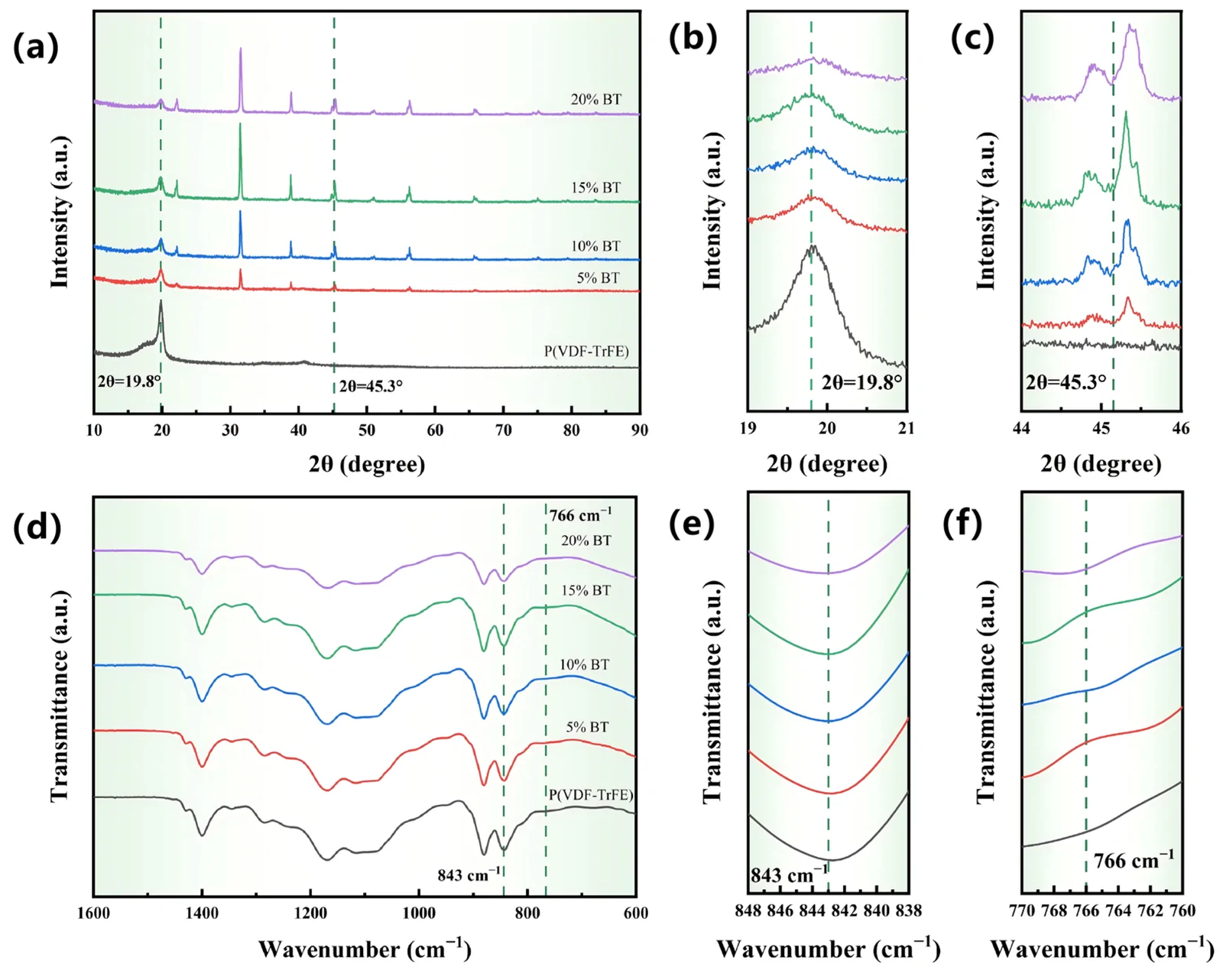
(**a**) XRD patterns for different loading concentrations; (**b**) enlarged view of the characteristic XRD peak of the β-phase in P(VDF-TrFE); (**c**) enlarged view of the characteristic peak of the BT tetragonal phase; (**d**) FTIR spectra for different loading concentrations; (**e**) enlarged view of the characteristic FTIR peak of the β-phase in P(VDF-TrFE); (**f**) enlarged view of the characteristic FTIR peak of the α-phase in P(VDF-TrFE).

To investigate the crystal structure in piezoelectric films, XRD analysis was performed over a 2θ range of 10°–90° at a scan rate of 8°/min. Figure 2a–c illustrate structural changes in the piezoelectric films at different loading concentrations. The reflection peak at 2θ = 19.8°, corresponding to the (100)/(210) planes, is characteristic of the polar β-phase of P(VDF-TrFE) [46]. The peak at 2θ = 45.3° corresponds to the split (002)/(200) crystal planes and is also a characteristic peak of BT [47]. To investigate the chemical structure of the piezoelectric films, FTIR analysis was performed on the films. The wavenumbers ranged from 600 cm^−1^ to 1600 cm^−1^, as shown in Figure 2d–f. The FTIR characteristic peaks were largely similar across different loadings, mainly because the functional groups in the films remained consistent. Additionally, the absorption band at 843 cm^−1^ is characteristic of the β-phase, whereas the peak at 766 cm^−1^ is attributed to the α-phase in the piezoelectric films (the XRD and FTIR of raw materials are shown in Figure S2) [48]. The β-phase content varies, reaching a maximum of 86.1% at a BT loading concentration of 15%. The content of the β-phase can be seen in the Supporting Information (Figure S3). Overall, both XRD and FTIR analyses conclusively demonstrate that the piezoelectric films prepared by this method exhibit a high content of the β-phase.

#### 3.1.2. SEM

**Figure 3:**
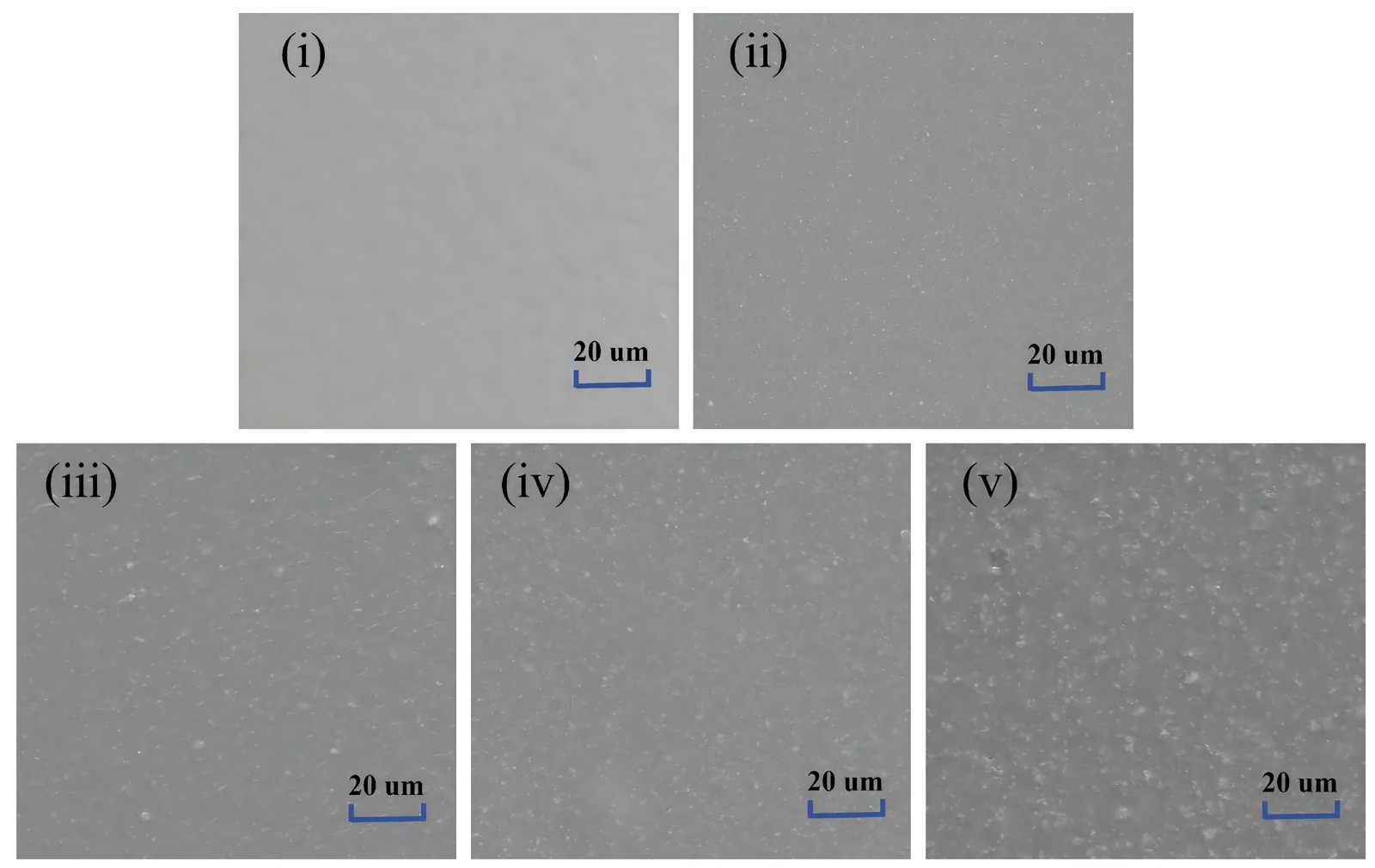
(**i**) Pure P(VDF-TrFE) film; (**ii**) 5% BT/P(VDF-TrFE) film; (**iii**) 10% BT/P(VDF-TrFE) film; (**iv**) 15% BT/P(VDF-TrFE) film; (**v**) 20% BT/P(VDF-TrFE) film.

SEM analysis was performed on the as-prepared piezoelectric films, as shown in Figure 3i–v. The images show that the BT nanoparticle content in the films increases significantly with increasing BT loading. When the BT loading reaches 20%, obvious particle agglomeration is observed on the film surface. This agglomeration adversely affects the material performance. The EDS patterns and elemental composition variations at different BT loadings are provided in Figure S4a–e.

### 3.2. Testing of the Electrical Properties of Materials

#### 3.2.1. Testing of Dielectric Constant–Frequency

**Figure 4:**
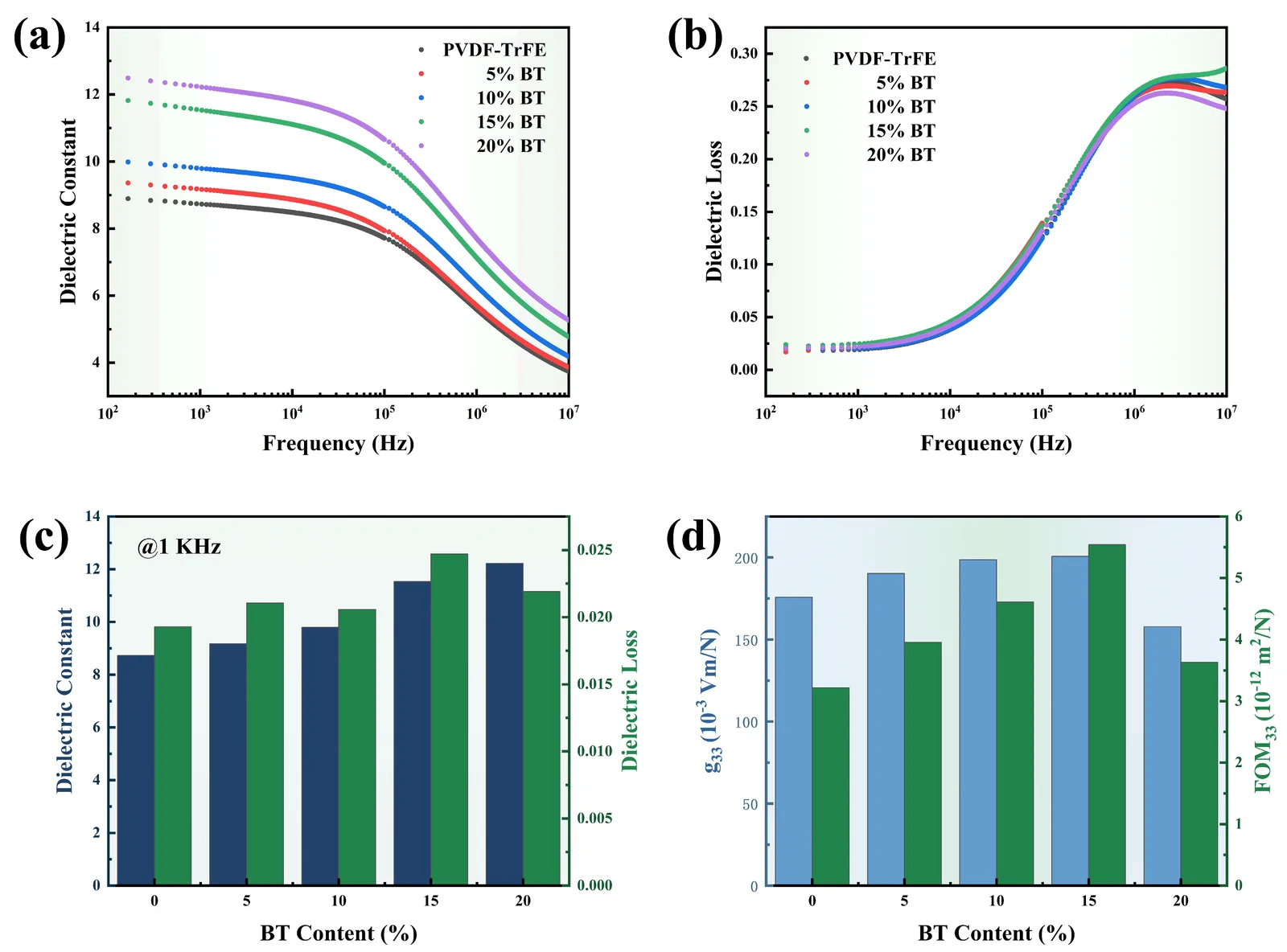
(**a**) Dielectric constant–frequency of the piezoelectric films; (**b**) dielectric loss-frequency of the piezoelectric films; (**c**) variation in the dielectric constant and dielectric loss of the piezoelectric films with different loading concentrations at 1 kHz; (**d**) g_33_ and FOM_33_ under different loading concentrations.

This study further investigates the dielectric property variations in the composite films with different BT loading concentrations, as shown in Figure 4a–d. Figure 4a depicts the frequency dependence of the dielectric constant under various loading conditions. Since the dielectric constant of BT is higher than that of P(VDF-TrFE), the dielectric constant increases with increasing loading concentration. Additionally, at higher frequencies, the motion of dipoles becomes more restricted [49]. Consequently, regardless of the loading concentration, the dielectric constant decreases with increasing frequency. Figure 4b displays the dielectric loss performance. The dielectric loss, which first increases and then decreases with rising frequency, is predominantly attributable to the dipolar relaxation inherent to the P(VDF-TrFE) [50]. At low frequencies, the electric field changes slowly, resulting in polarization that is nearly in phase with the electric field, thus yielding low loss. As frequency increases, the phase difference gradually widens, leading to progressively higher loss. In the high-frequency region, the phase difference between dipole reorientation and electric field changes becomes too large. At this point, the loss due to phase difference decreases, with the main contribution coming from electronic and ionic polarization, thus reducing the dielectric loss. Figure 4c directly illustrates the relationship between the dielectric constant and dielectric loss of different loading concentrations at a frequency of 1 kHz. The dielectric constant increases as the BT loading concentration rises. Dielectric loss initially increases but then decreases when the concentration reaches 20%. This phenomenon may be partially attributed to the formation of a physical barrier by high-concentration BT nanoparticles, which restricts the movement of P(VDF-TrFE) molecular chains and pins ferroelectric domains. Additionally, the increased filler loading may alter the interfacial structure and reduce leakage current pathways, thereby contributing to the observed slight reduction in dielectric loss. However, the exact mechanism warrants further investigation with direct leakage current measurements [51]. To investigate the film’s sensing and energy harvesting performance, as shown in Figure 4d, it can be seen that both g_33_ and FOM_33_ reach their maximum values at a loading concentration of 15%, indicating that 15% yields the best sensing and energy harvesting performance. Also, when the loading resistance reached 4 × 10^6^ Ω, the maximum power density reached 3.83 mW/m^2^ (detailed power density data are shown in Figures S6 and S7), which is comparable to those of previously reported composite-based nanogenerators fabricated by electrospinning or solution-casting methods. 

#### 3.2.2. Testing of Dielectric Constant-Temperature

**Figure 5:**
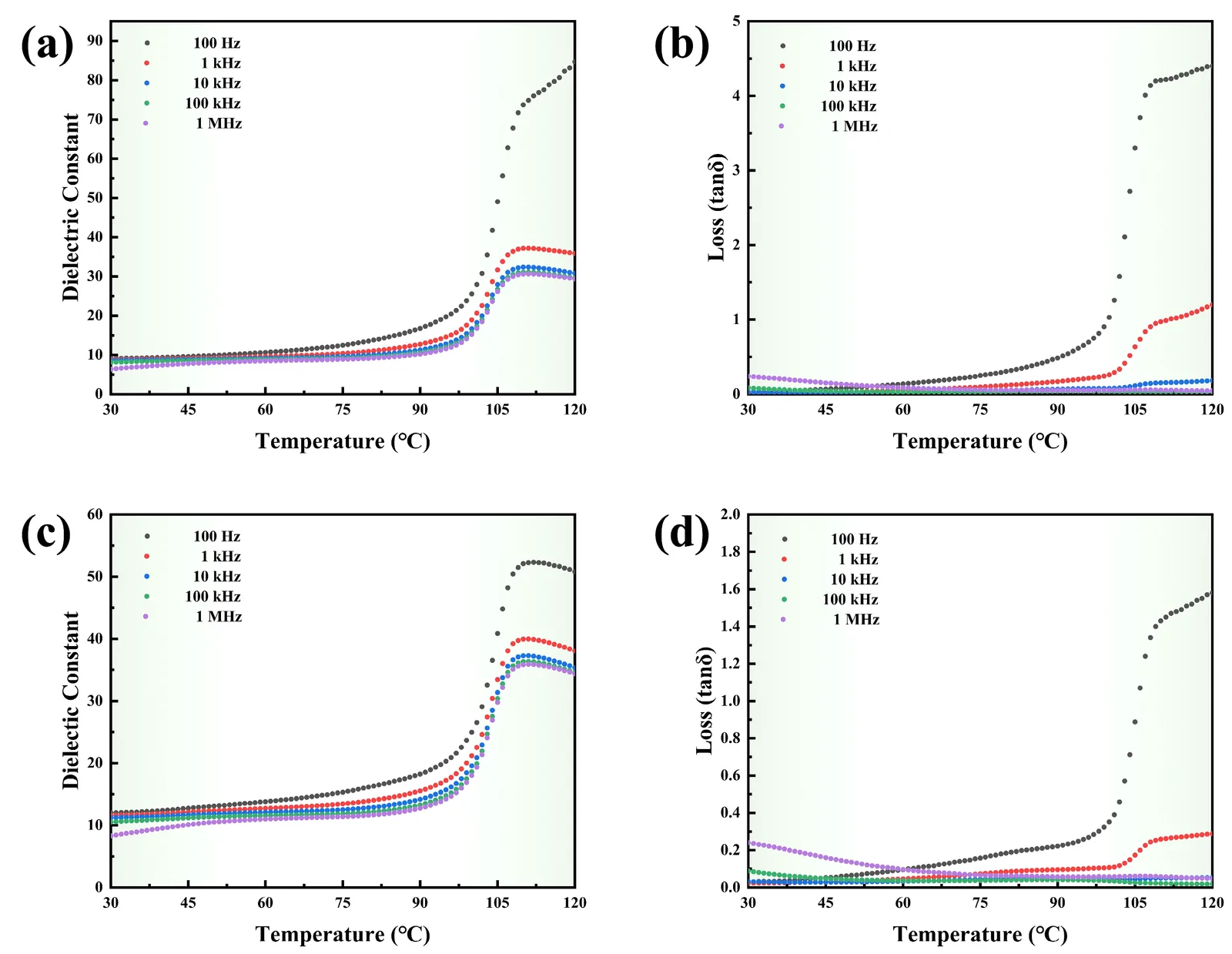
(**a**) Dielectric constant temperature of the pure P(VDF-TrFE) film; (**b**) dielectric loss temperature of the pure P(VDF-TrFE) film; (**c**) dielectric constant temperature of the 15% BT/P(VDF-TrFE) film; (**d**) dielectric loss temperature of the 15% BT/P(VDF-TrFE) film.

Dielectric temperature-dependent measurements were performed to further investigate the dielectric constant and dielectric loss of the piezoelectric films at various temperatures and frequencies. The dielectric temperature spectra and corresponding dielectric losses of the unloaded film and the 15% BT-loaded film are compared in Figure 5 (data for other loading concentrations are provided in Supporting Information Figure S5). As shown in Figure 5a,c, regardless of whether BT is loaded, the dielectric constant decreases at high frequencies under the same temperature conditions. This decrease is consistent with the explanation in the dielectric-frequency spectrum, as shown in Figure 4. At the same frequency, prior to the Curie temperature (Tc), the dielectric constant increases relatively slowly with rising temperature. This is primarily because temperature increases below Tc do not significantly affect the material’s ferroelectric domain structure, resulting in only a gradual rise in the dielectric constant. As temperature gradually approaches Tc, the ferroelectric domain structure begins to undergo extensive breakdown. Dipoles transition from ordered to disordered states, resulting in a rapid increase in the dielectric constant across all frequencies, culminating in a peak. Subsequently, as temperature continues to rise, the ferroelectric phase structure almost completely transforms into a paraelectric phase, leading to a slight decrease in the dielectric constant [52]. Figure 5b,d illustrates dielectric loss under varying temperature and frequency conditions. At low frequencies, increased temperature enhances polymer chain mobility, facilitating the response of dipole orientation and interfacial polarization to electric fields. This leads to increased polarization relaxation and dissipation, causing dielectric loss to rise with temperature. At high frequencies, however, the electric field reversal rate far exceeds the dipole response speed. Temperature elevation intensifies thermal agitation, making it difficult for dipoles to synchronously follow electric field changes. This weakens relaxation loss, causing dielectric loss to decrease with increasing temperature [53]. Furthermore, compared to the neat P(VDF-TrFE) film, the 15% BT-loaded film exhibits a slight upshift in Tc at a given frequency (Figure 5; complete data for other loading concentrations are provided in Figure S5). This trend suggests that the incorporation of BT nanoparticles may restrict polymer chain mobility near the interfacial regions, thereby elevating the energy barrier for ferroelectric-to-paraelectric transition [54].

#### 3.2.3. Testing of Ferroelectric Properties

**Figure 6:**
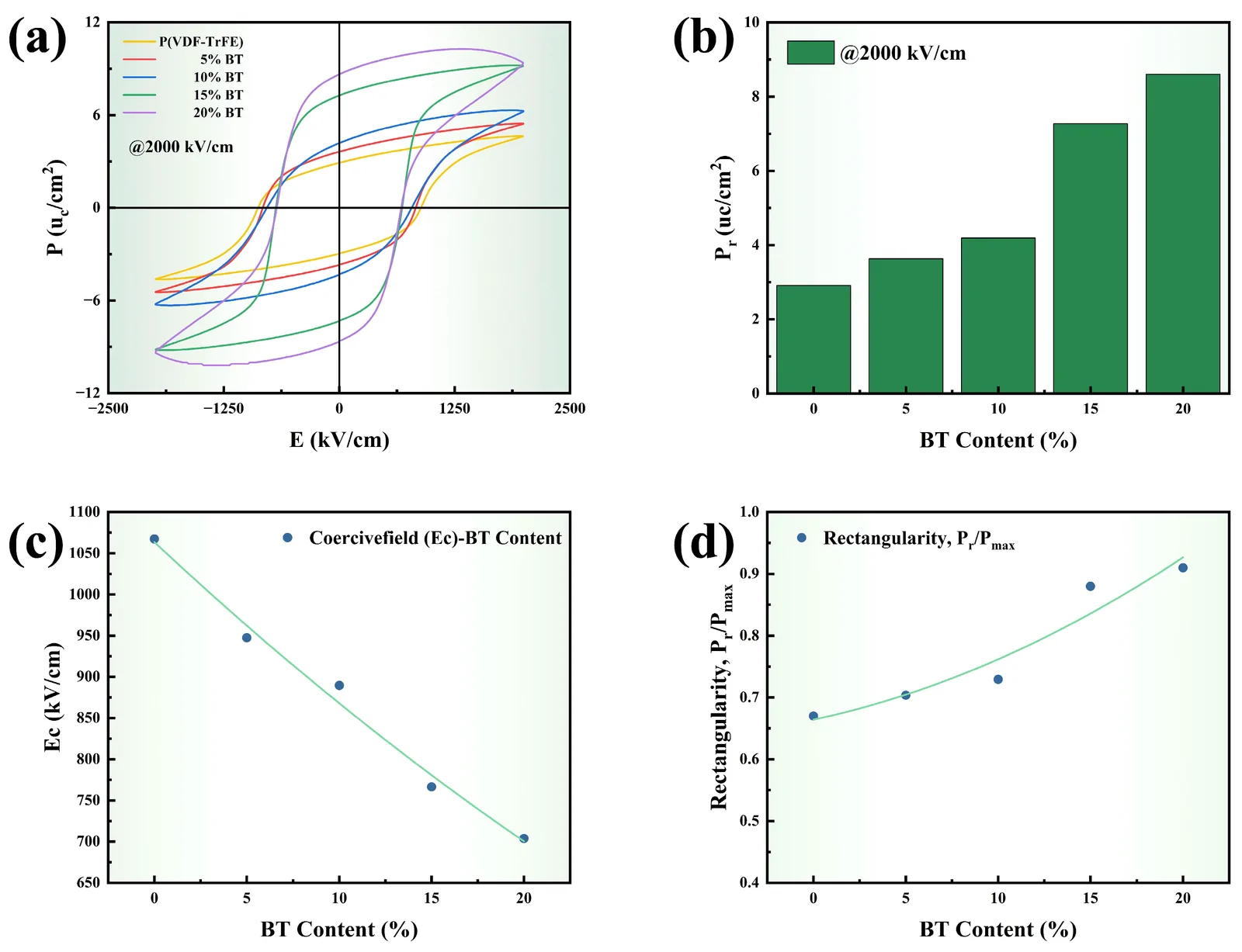
(**a**) P-E curves at an electric field of 2000 kV/cm for different loading concentrations; (**b**) trends in remanent polarization intensity under an electric field of 2000 kV/cm; (**c**) coercive electric fields of piezoelectric films at different loading concentrations; (**d**) ferroelectric rectangularity of piezoelectric films at different loading concentrations.

To further investigate the ferroelectric properties of the piezoelectric films, ferroelectric measurements were conducted, as shown in Figure 6. Figure 6a shows the P-E curves at an electric field of 2000 kV/cm for different loading concentrations. The P-E curves for all concentrations are highly symmetrical, indicating that the films with all loading concentrations exhibit excellent ferroelectric response [55]. Consequently, both maximum polarization and remanent polarization intensities exhibit an upward trend with BT loading. Figure 6b shows the variation in remanent polarization intensity at different loading concentrations under an applied electric field of 2000 kV/cm. Figure 6c shows the variation in coercive field with loading concentration. The coercive field gradually decreased with increasing BT loading, primarily because the coercive field of BT is lower than that of P(VDF-TrFE). Figure 6d shows the rectangularity (ratio of remanent polarization intensity to maximum polarization intensity) at different loading concentrations. Owing to the excellent ferroelectric properties of BT, the squareness increased with increasing BT loading.

#### 3.2.4. Testing of Piezoelectric Properties

**Figure 7:**
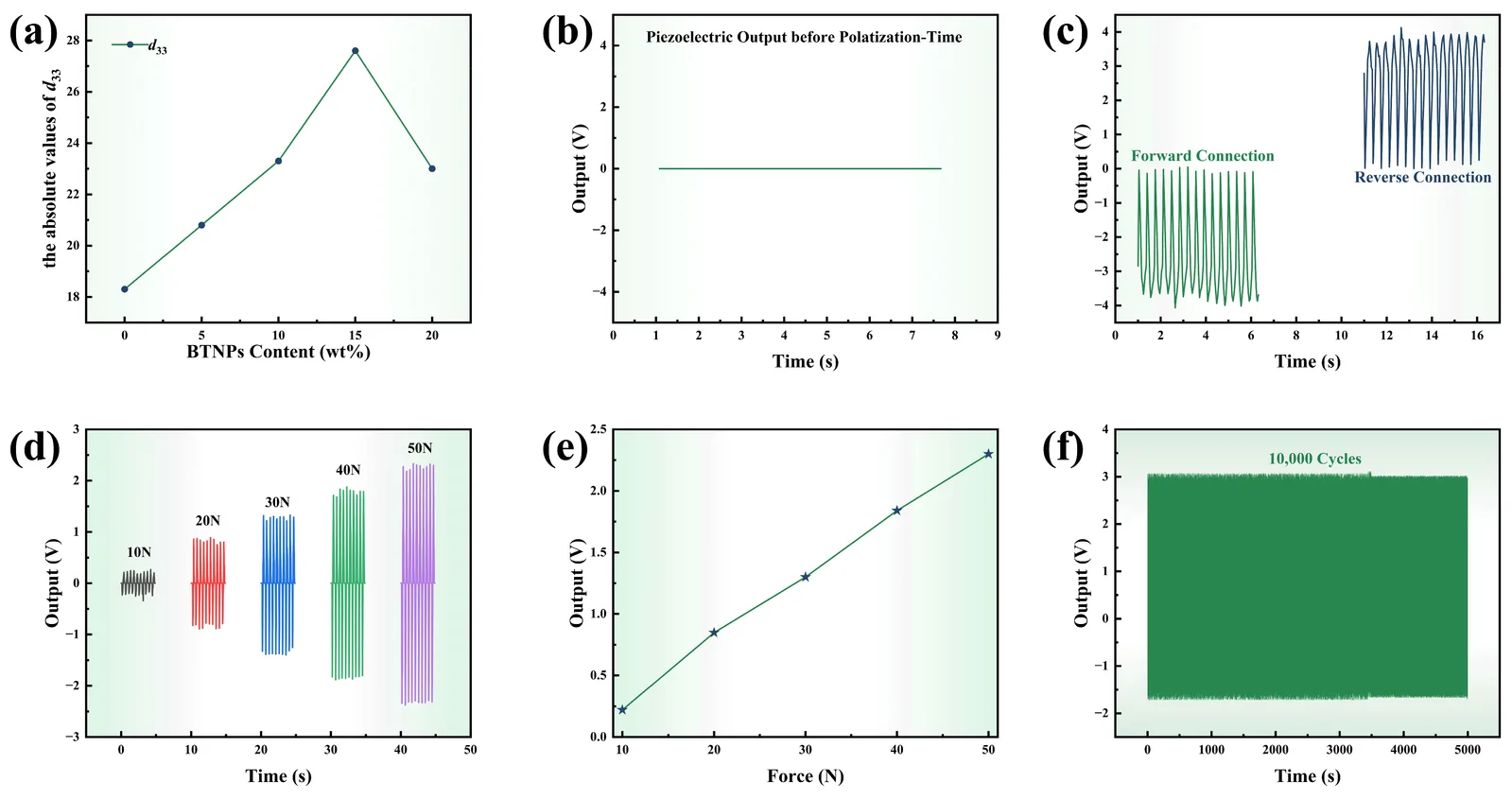
(**a**) Variation in the absolute value of d_33_ at different loading concentrations; (**b**) piezoelectric output time before polarization; (**c**) piezoelectric output voltage as a function of time under forward and reversed electrode connections; (**d**) piezoelectric output under different forces; (**e**) the linear relationship between different forces and piezoelectric output; (**f**) piezoelectric output cycle life test (10,000 cycles).

As shown in Figure 7, to systematically investigate the sensing performance under varying filler loadings, the as-prepared piezoelectric composite films with different concentrations were subjected to corona poling. Prior to poling, a gold electrode was sputter-deposited onto one side of each film to serve as the bottom electrode and facilitate charge injection. Based on the optimization of poling conditions (Supporting Information, Figures S8 and S9), the corona poling was deliberately conducted at 75 °C and 16 kV, as these parameters were determined to maximize the absolute value of the d_33_ coefficient, thereby ensuring the optimal piezoelectric response for subsequent sensing characterization. The polarized piezoelectric films were then left at room temperature for 24 h to reduce the influence of residual surface charge on the d_33_ measurements. The d_33_ values for different loading concentrations are plotted in Figure 7a (the raw d_33_ values are negative; the actual measured negative d_33_ values are presented in Figure S10). The absolute value of the piezoelectric coefficient, |d_33_|, first increased and then decreased with rising BT loading. In the low-loading regime, the increase in |d_33_| primarily stems from the nucleation effect of BT, which promotes β-phase crystallization in the P(VDF-TrFE) matrix and thereby enhances the matrix contribution. It should be noted that the intrinsic piezoelectric response of BT is opposite in sign to that of the poled matrix; however, its magnitude is far smaller than the β-phase-induced enhancement of the matrix, so that the overall |d_33_| remains dominated by the matrix contribution and increases with loading. At excessively high loadings, however, agglomeration of BT nanoparticles impedes piezoelectric charge transfer, causing |d_33_| to decline. These results indicate that the composition-dependent |d_33_| reflects a competition between the nucleation-induced enhancement of the matrix and the agglomeration-induced deterioration of charge transport, with an optimal BT loading balancing the two effects. Since the |d_33_| value peaks at a BT loading level of 15%, this concentration was employed throughout subsequent piezoelectric output and cyclic life testing. To eliminate triboelectric interference on piezoelectric output during testing, comparisons were made between output states before and after polarization, as illustrated in Figure 7b,c. In addition, we performed a control experiment under preload. Moreover, Figure 7c illustrates the time-domain piezoelectric output characteristics measured after poling, comparing the responses under forward and reverse electrode polarities. Without polarization, the piezoelectric output exhibits a low peak, primarily due to environmental noise. Following poling, the sensor delivers stable piezoelectric output under applied force. When the electrode connection is reversed, the output voltage exhibits a clear polarity reversal. This confirms that the output voltage originated from the piezoelectric effect rather than from triboelectric contributions. We further investigated the piezoelectric output under varying applied forces and analyzed the linear relationship between the output voltage and the applied force, as presented in Figure 7d,e. Specifically, a piezoelectric output of 0.221 V was achieved under an applied force of 10.0 N, 0.848 V at 20.0 N, 1.30 V at 30.0 N, 1.84 V at 40.0 N, and 2.30 V at 50 N. The sensitivity of this sensor was approximately 0.0515 V/N. Furthermore, the long-term cyclic stability of the sensor was systematically evaluated. The piezoelectric output exhibited a slight degradation of approximately 3% after 10,000 continuous loading cycles under 50 N. This marginal decline is primarily attributed to the relaxation of oriented polymer chains and the gradual decay of interfacial polarization under prolonged mechanical fatigue. In flexible polymer–ceramic composites, such minor output attenuation is inevitable due to the viscoelastic nature of the polymer matrix and the gradual redistribution of space charges at the filler–matrix interfaces. Importantly, the 3% degradation falls well within the acceptable tolerance for most wearable sensing applications, confirming that the tape-cast dense composite films possess sufficient durability and reliability for continuous human motion monitoring.

**Figure 8:**
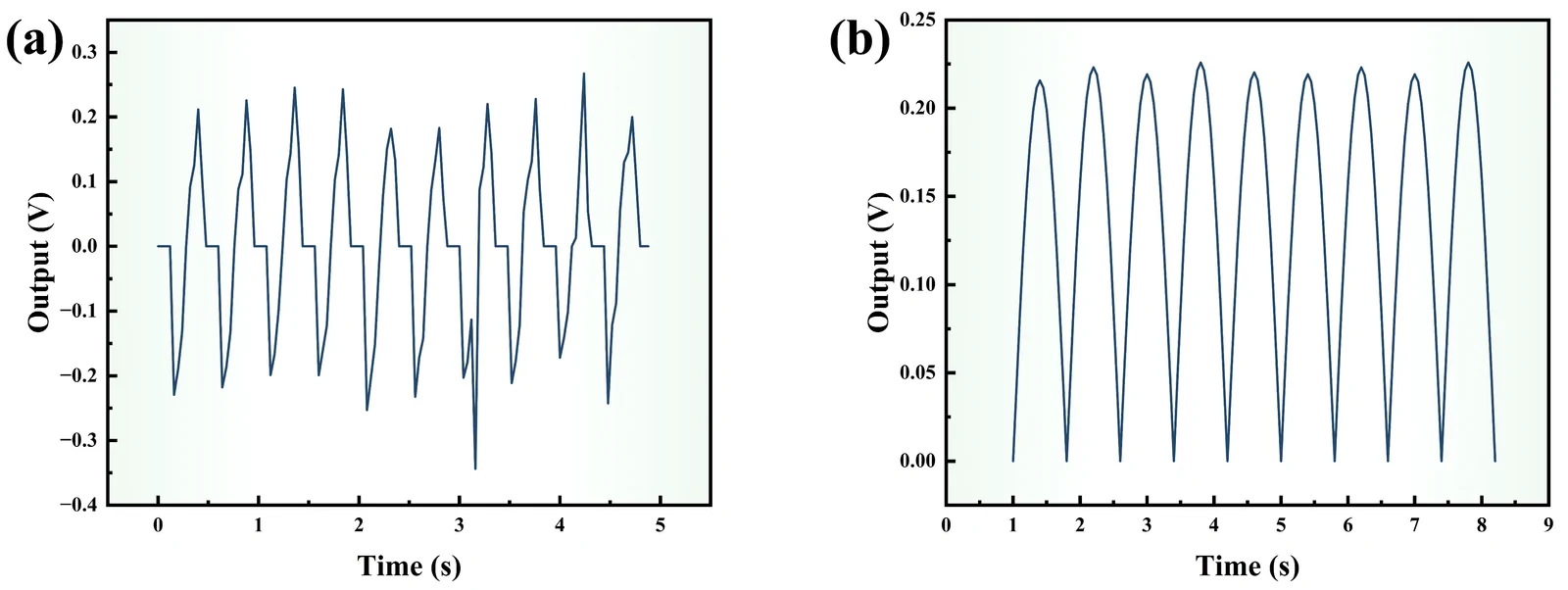
(**a**) Piezoelectric output under impact loading; (**b**) piezoelectric output under compressive semi-sinusoidal loading.

To further substantiate the validity of the impact loading method employed for piezoelectric output measurements in this study, two control experiments were conducted: an impact test with a maximum applied load of 10 N and a compressive semi-sinusoidal loading test with a peak compressive force of 10 N (the piezoelectric output under a preload-only condition, as shown in Figure S11). As shown in Figure 8, the piezoelectric outputs obtained under the two loading protocols are nearly identical, with a peak voltage of approximately 0.22 V. This agreement can be rationalized by the piezoelectric constitutive relation Q = d_33_·F: under the present testing conditions, the generated charge is governed primarily by the instantaneous force and is essentially independent of the loading mode and loading rate. Since the peak force is 10 N in both cases, the peak charge is identical; assuming a nearly constant equivalent capacitance of the device, the peak voltage is also identical, approximately 0.22 V. These results demonstrate that the device exhibits a linear and repeatable response within the 10 N loading range. In addition, the polarity difference between the voltage waveforms under the two loading protocols is consistent with the respective loading processes. Under compressive semi-sinusoidal loading, the presence of a preload ensures that the force remains compressive throughout, so the output voltage retains the same polarity and fluctuates only between 0 and 0.22 V. Under impact loading, however, unloading is accompanied by rebound and vibration, such that the stress crosses zero and generates charges of opposite sign, thereby producing negative peaks. The negative peak amplitude is comparable to that of the positive peak, indicating that most of the elastic energy stored during compression is released during rebound and that the contact behavior is predominantly elastic with low energy dissipation.

### 3.3. COMSOL Finite Element Simulation

**Figure 9:**
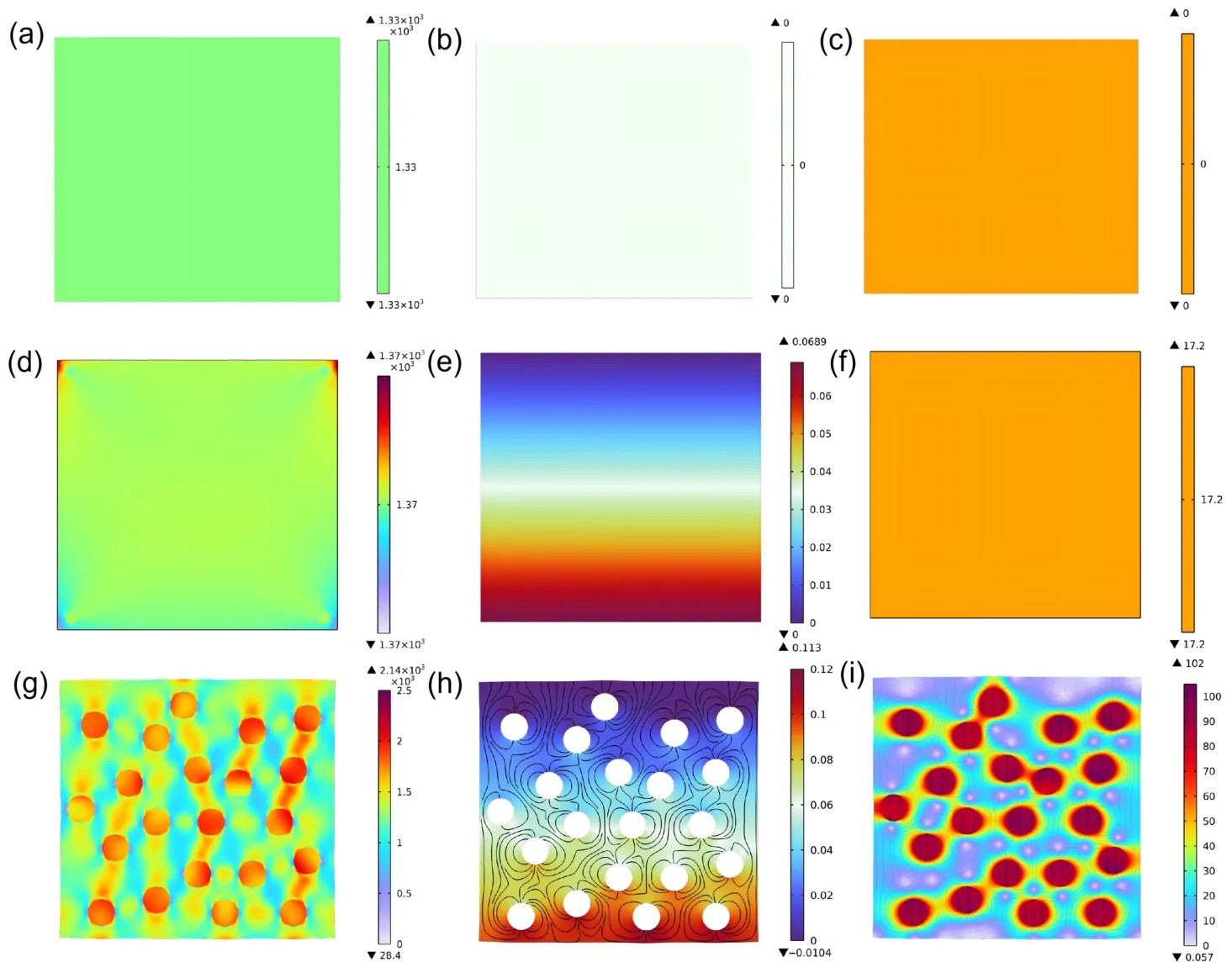
(**a**) Stress distribution of P(VDF-TrFE); (**b**) piezoelectric potential of P(VDF-TrFE); (**c**) electric field distribution of P(VDF-TrFE); (**d**) stress distribution of BT nanoparticles; (**e**) piezoelectric potential of BT nanoparticles; (**f**) electric field distribution of BT nanoparticles; (**g**) stress distribution of 15% BT/P(VDF-TrFE); (**h**) piezoelectric potential of 15% BT/P(VDF-TrFE); (**i**) electric field distribution of 15% BT/P(VDF-TrFE).

As shown in Figure 9, the finite element analysis conducted using COMSOL 5.4 software is intended to investigate the distribution of stress, potential, and electric fields in P(VDF-TrFE), BT, and piezoelectric films with different loading concentrations. Except for 0% and 15%, the results for all other loading conditions are presented in Figure S12 in the Supplementary Information. The potentials generated within this model were as follows: 0, 0.066, 0.093, 0.125, 0.147, and 0.162 mV. To investigate the rate of change, an additional simulation was performed with a loading level of 25%. Based on these data, the growth rate drops significantly between 15% and 20% loading. The growth rate can be directly determined by plotting the slope using this data set. Simply put, the improvement in piezoelectric performance due to the volume fraction (solid content) becomes negligible after 15%. However, the introduction of solid content inevitably leads to an increase in bubbles and impurities—the more solid content added, the greater the contamination. Therefore, after 15%, the performance gain from the solid content is outweighed by the performance loss caused by impurities, resulting in a decline in performance. The reason is that under the same force, pure BT has higher hardness, resulting in smaller deformation. In contrast, the structure of the P(VDF-TrFE) composite compresses the BT particles due to the difference in Young’s modulus, leading to stress concentration and greater deformation, which in turn enhances the piezoelectric performance. As shown in the electric field strength graph, the higher the solid content, the more pronounced the field concentration becomes. This merely indicates a higher susceptibility to breakdown, which is consistent with the results observed during ferroelectric testing.

### 3.4. Practical Applications

**Figure 10:**
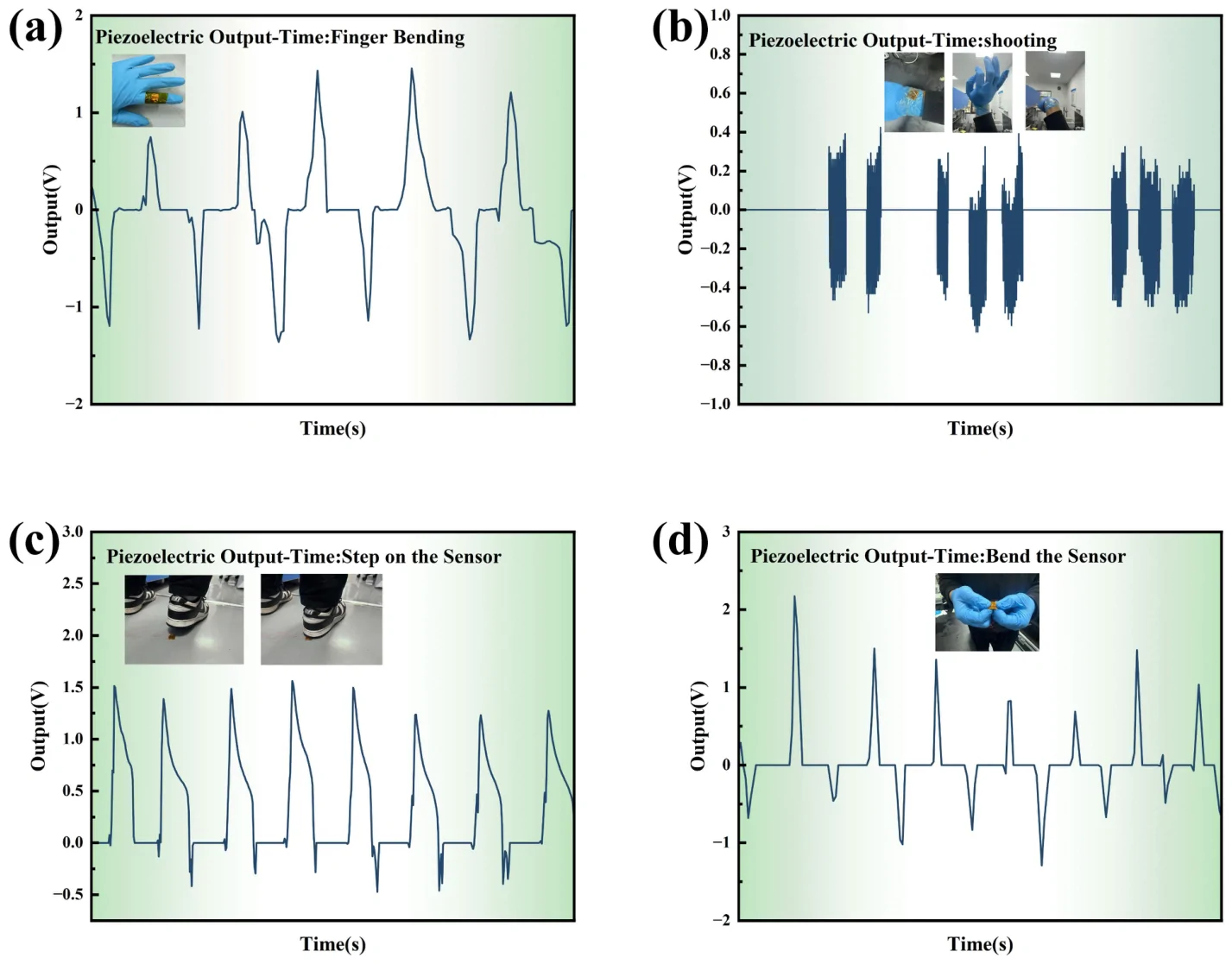
(**a**) Finger bending; (**b**) shooting stance; (**c**) foot press; (**d**) sensor bending.

To investigate the performance of the sensor developed in this paper for monitoring human joint movements and external forces, practical application tests were conducted. As shown in Figure 10a–d, distinct piezoelectric outputs are generated under different joint motion patterns and external force stimuli. This demonstrates that the piezoelectric sensor can respond to external stimuli under various conditions.

## 4. Discussion

This study fabricated BT/P(VDF-TrFE) piezoelectric films via the tape casting method. It was demonstrated that the incorporation of BT nanoparticles effectively promotes the crystallization of the highly piezoelectric β-phase in P(VDF-TrFE), as BT particles serve as heterogeneous nucleation sites that facilitate β-phase formation, while their own piezoelectric contribution is minor and opposite in sign to that of the poled matrix, playing a negligible role in the overall piezoelectric output. XRD and FTIR analyses revealed that the relative fraction of the β-phase first increased and then decreased with increasing BT content, which was consistent with the evolution of the piezoelectric coefficient d_33_. The piezoelectric performance was improved at low filler loadings but deteriorated at excessive contents, mainly due to severe particle agglomeration and increased interfacial stress. The optimal performance was achieved at 15 wt% BT loading, with a d_33_ value of 27.6 pC/N, remarkably higher than that of pure P(VDF-TrFE) (18.3 pC/N). By comparing the output signals of polarized and unpolarized samples, the contribution of the triboelectric effect was excluded, verifying that the electrical response mainly originated from the piezoelectric effect. From the force–output relationships, the sensor sensitivity at 15 wt% BT loading was determined to be approximately 0.0515 V/N. Under an applied force of 50 N, the composite film sensor delivered an output voltage of approximately 2.3 V. The sensor was further utilized for human motion monitoring, and four typical movement patterns were tested. The results showed distinguishable output amplitudes and waveforms corresponding to different motion states, which provides a basis for subsequent motion recognition combined with machine learning algorithms. In addition, the dielectric properties, ferroelectric behavior, polarization strength, and cycling stability of the films were systematically characterized. Overall, this work provides a feasible and straightforward strategy for constructing high-performance piezoelectric sensors, showing promising potential in wearable electronics and human–machine interaction.

## Figures and Tables

**Table 1:** Amount of BT used in preparing films of different concentrations.

Content (%)	DMF (g)	P(VDF-TrFE) (g)	BaTiO_3_ (g)
0	7.2	0.8	0
5	7.2	0.76	0.04
10	7.2	0.72	0.08
15	7.2	0.68	0.12
20	7.2	0.64	0.16

## Data Availability

The original contributions presented in this study are included in the article/Supplementary Material. Further inquiries can be directed to the corresponding author.

## Supplementary Materials

The following supporting information can be downloaded at: https://www.mdpi.com/article/10.3390/polym18202518/s1.
