# Supplementary material for: Tape-Casting BaTiO3/P(VDF-TrFE) Piezoelectric Composite Films with Optimized Ferroelectric–Dielectric Performances for Wearable Human Motion Monitoring
Source: Polymers (Basel). 2026 Oct 14;18(20):2518. doi: 10.3390/polym18202518 (PMC13708904; doi:10.3390/polym18202518)
Supplement: Supplementary file 1 [file polymers-18-02518-s001.zip › polymers-4515105-supplementary.pdf]

SUPPORTING INFORMATION

Corresponding Author: Xi Yuan

Email: xiyuan@csu.edu.cn

Tape-Casting $BaTiO_3$/P(VDF-TrFE) Piezoelectric Composite Films with Optimized Ferroelectric-Dielectric Performances for Wearable Human Motion Monitoring

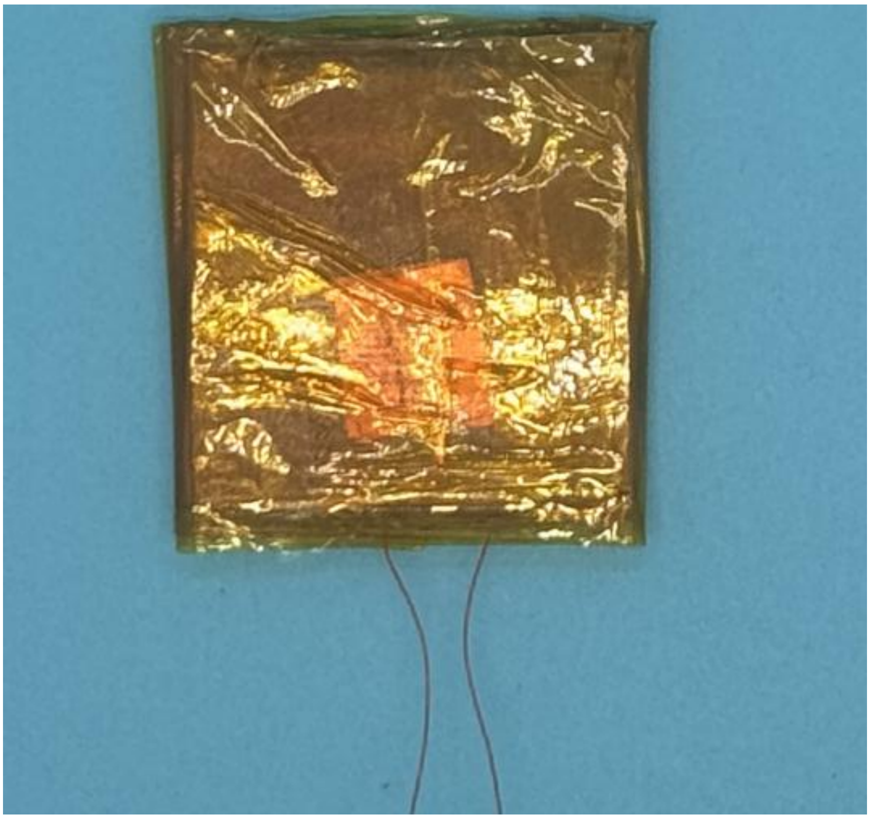

Figure S1. Image of the fabrication and assembly of a piezoelectric sensor.

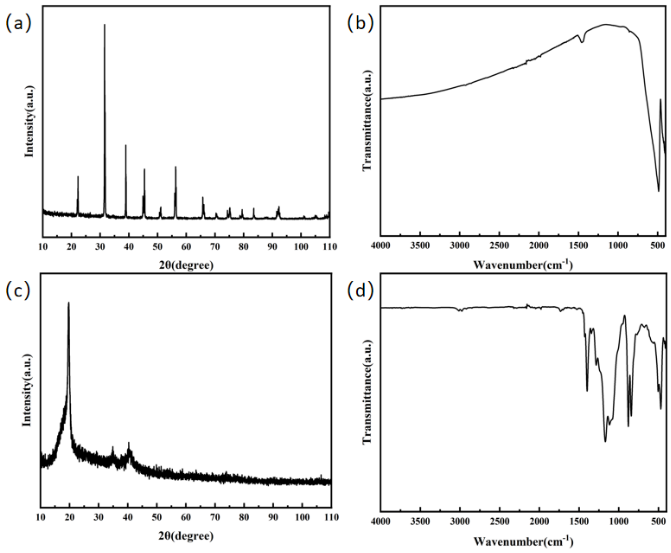

Figure S2. (a) The XRD of BaTiO3; (b) The FTIR of BaTiO3; (c) The XRD of P(VDF-TrFE); (d) The FTIR of P(VDF-TrFE).

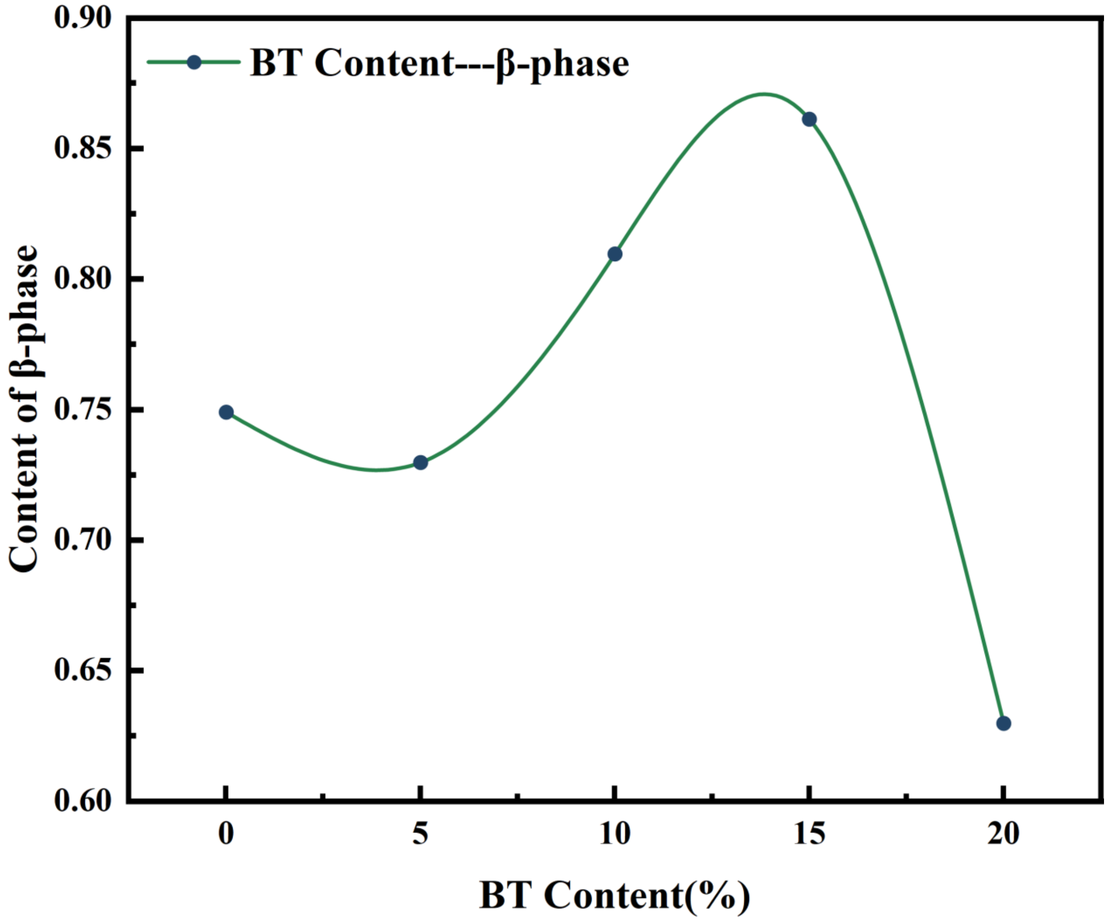

Figure S3. β-phase content with different BT concentration.

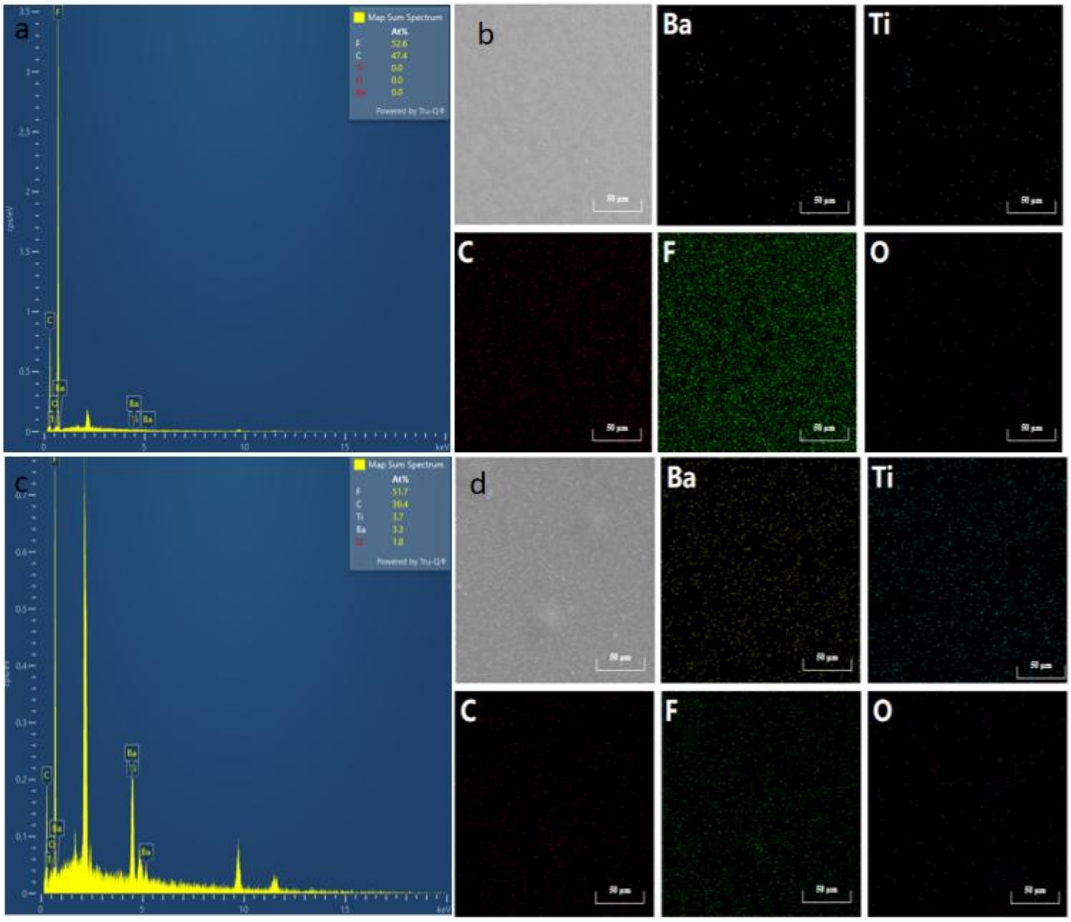

Figure S4-a. (a) Elemental content of C、F、O、Ba、Ti of the pure P(VDF-TrFE) film; (b) EDS image of the pure P(VDF-TrFE) film; (c) Elemental content of C、F、O、Ba、Ti of the 15% BT/P(VDF-TrFE) film; (d) EDS image of the 15% BT/P(VDF-TrFE) film.

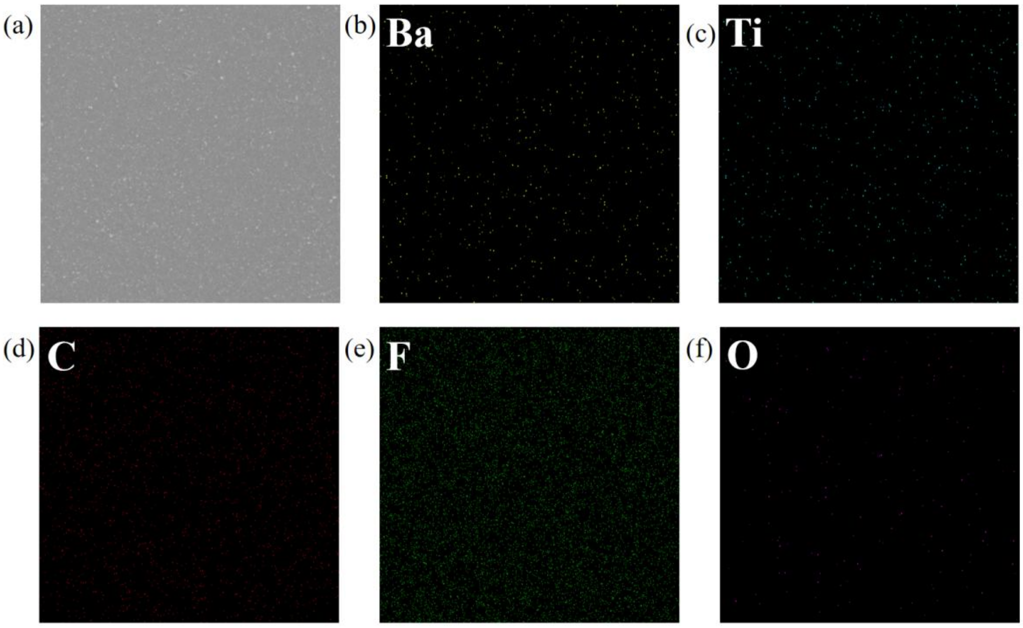

Figure S4-b. EDS at a doping concentration of 5%

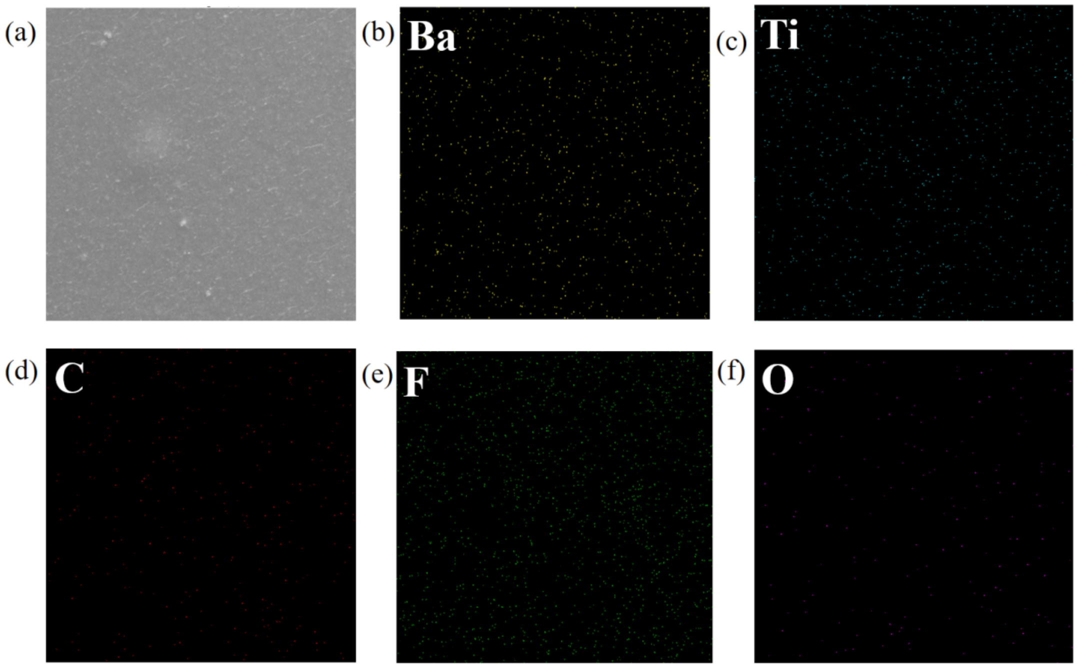

Figure S4-c. EDS at a doping concentration of 10%

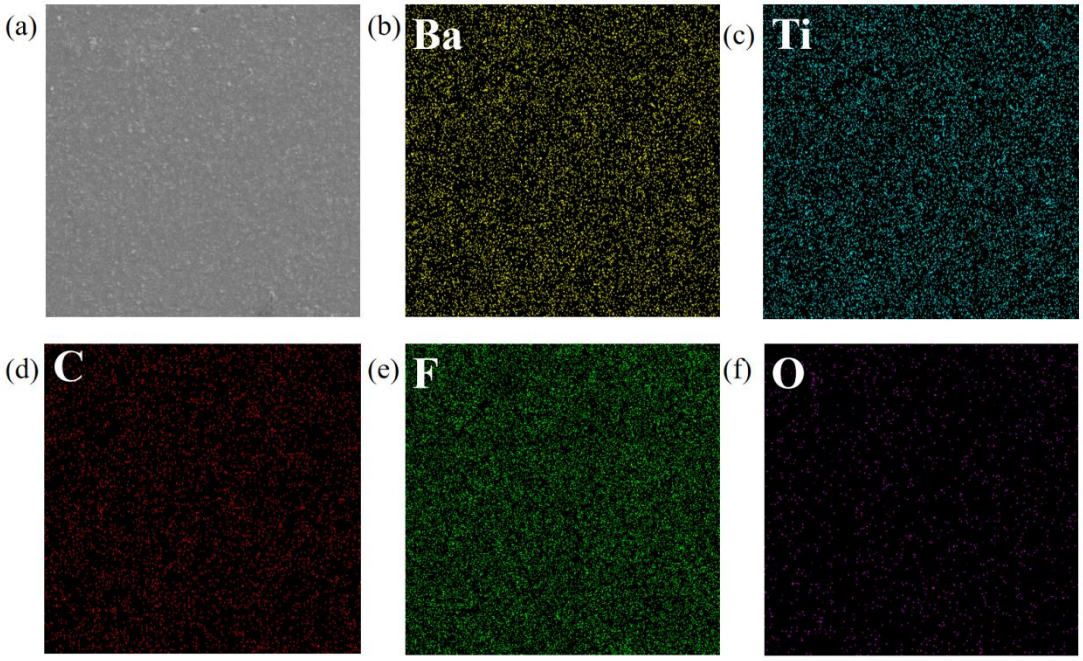

Figure S4-d. EDS at a doping concentration of 20%

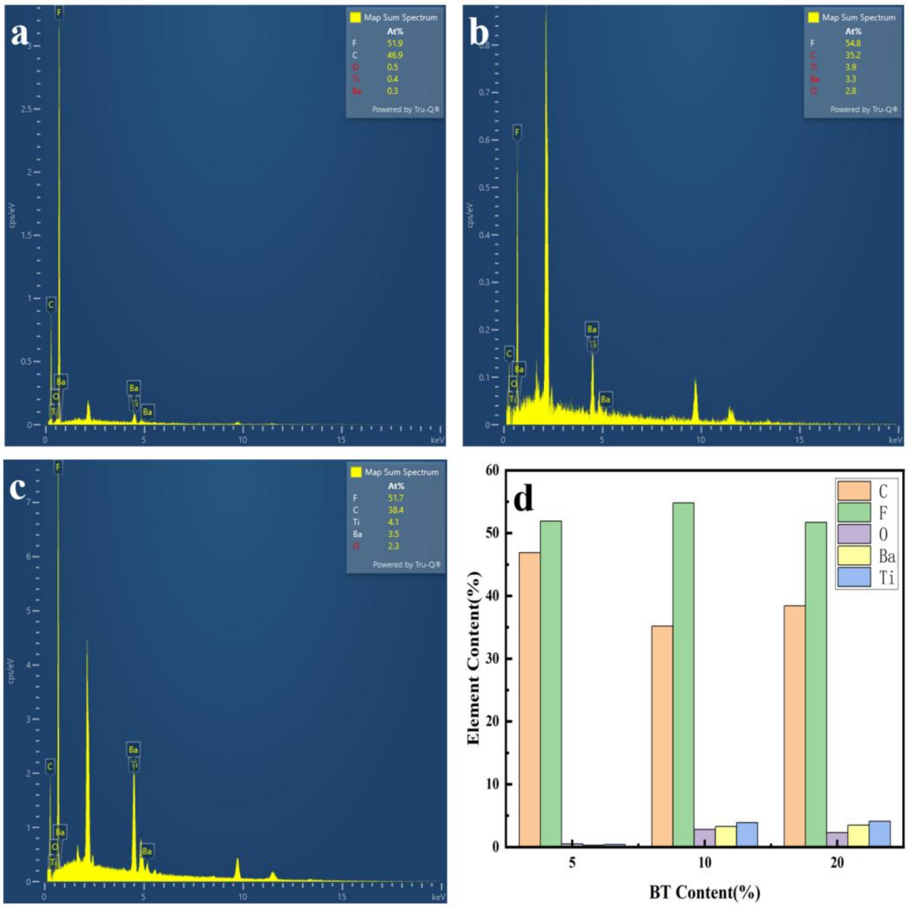

Figure S4-e. (a) Graph of Element Concentration at a 5% Doping Concentration; (b) Graph of Element Concentration at a 10% Doping Concentration; (c) Graph of Element Concentration at a 20% Doping Concentration; (d) Concentration trends for the five elements C, F, O, Ba, and Ti

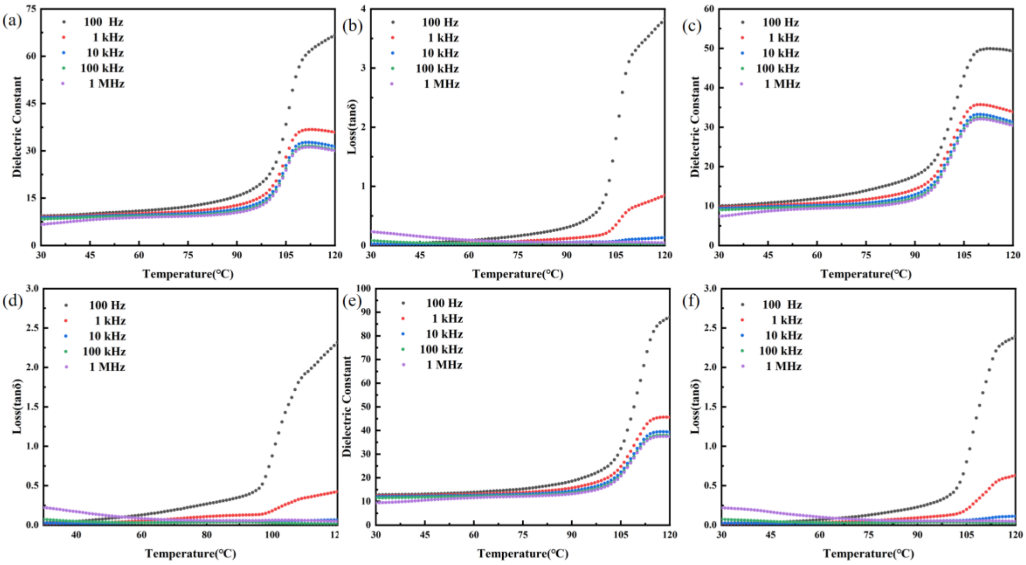

Figure S5. (a) Dielectric constant versus temperature at a doping concentration of 5%; (b) Dielectric loss versus temperature at a doping concentration of 5%; (c) Dielectric constant versus

temperature at a doping concentration of 10%; (d) Dielectric loss versus temperature at a doping concentration of 10%; (e) Dielectric constant versus temperature at a doping concentration of 20%; (f) Dielectric loss versus temperature at a doping concentration of 20%;

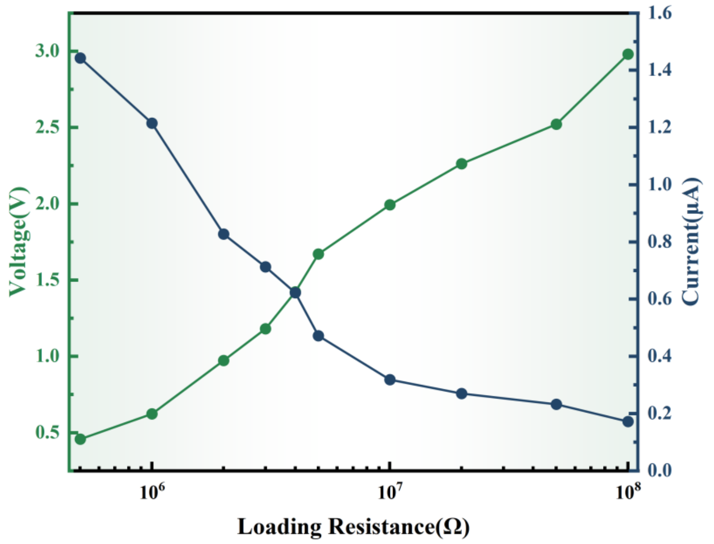

Figure S6. Output voltage and current characteristics of the piezoelectric device measured at various loading resistances.

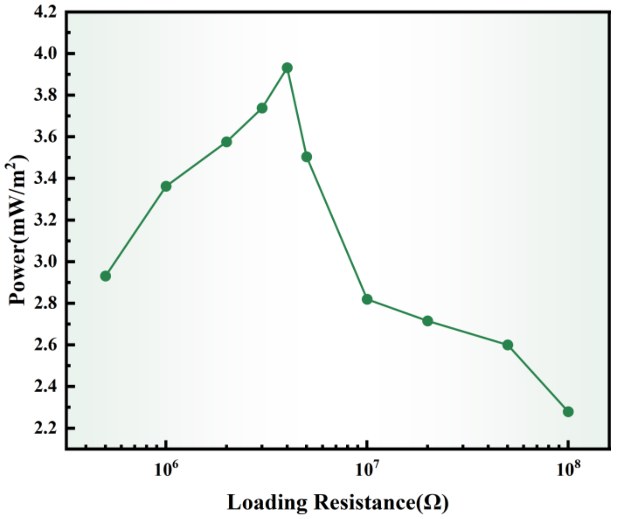

Figure S7. Output power density of the piezoelectric energy harvester measured at various loading resistances.

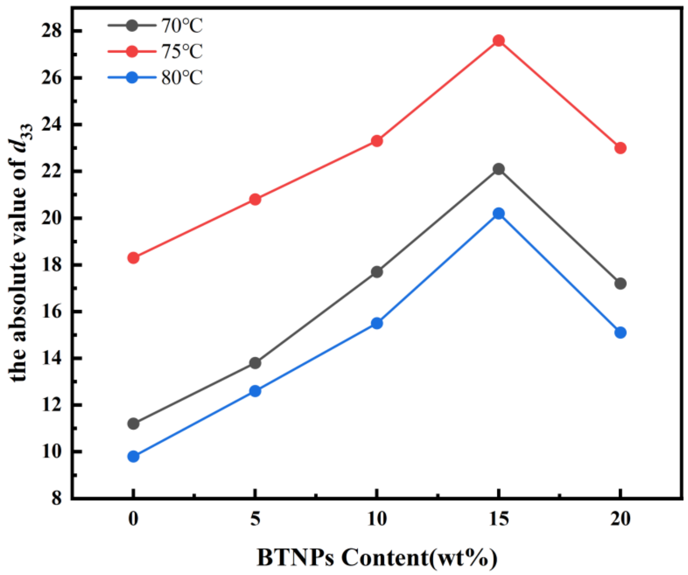

Figure S8. Variation of the absolute value d_{33} coefficient with poling temperature.

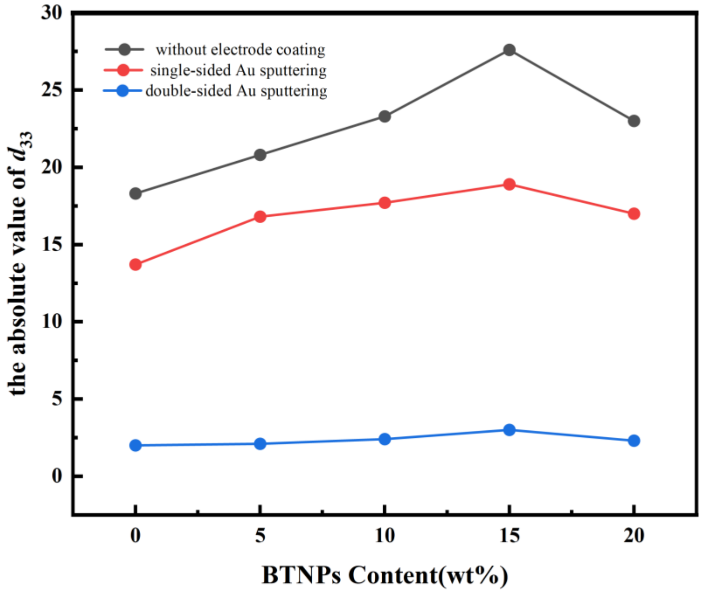

Figure S9. Variation of the absolute value d_{33} coefficient under different gold electrode deposition conditions.

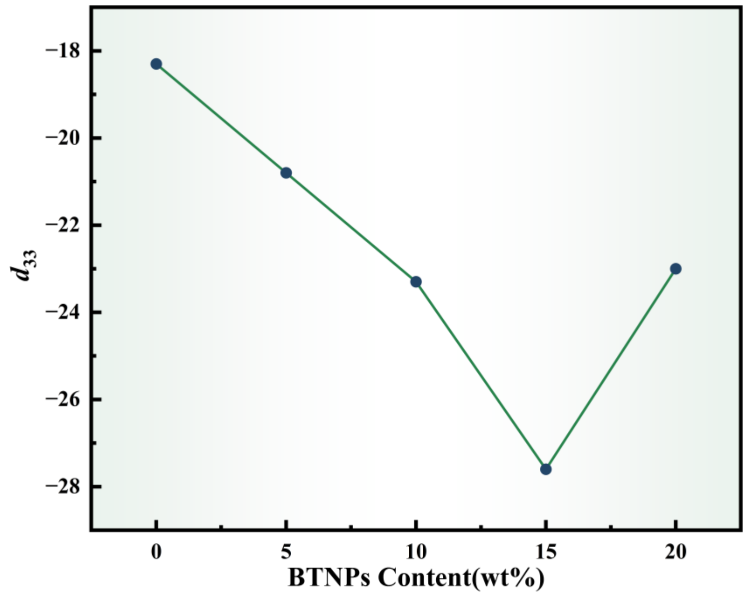

Figure S10. The raw data of d_{33} coefficient

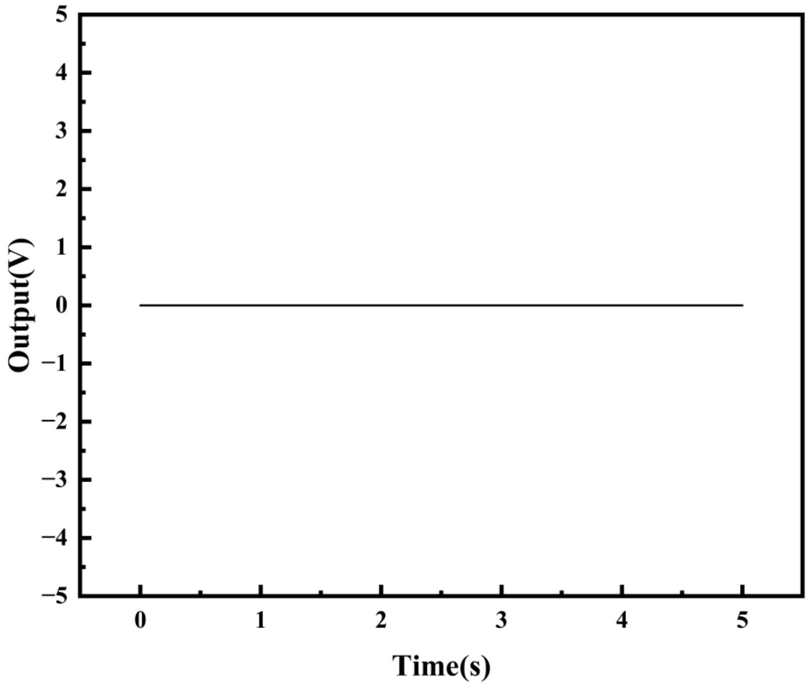

Figure S11. The output signal by only applying a small preload without other dynamic forces.

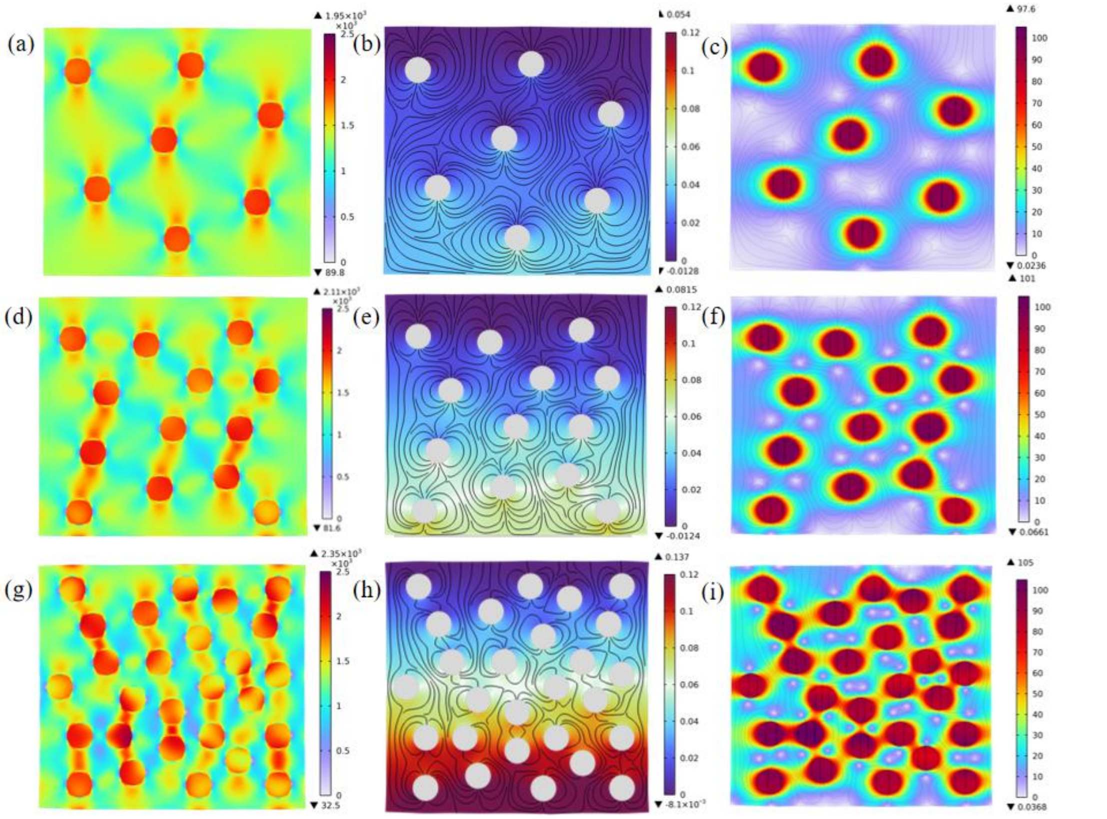

Figure S12. (a) Stress distribution of 5% BT/P(VDF-TrFE). (b) Piezoelectric potential of 5% BT/P(VDF-TrFE). (c) Electric field distribution of 5% BT/P(VDF-TrFE). (d) Stress distribution of 10% BT/P(VDF-TrFE). (e) Piezoelectric potential of 10% BT/P(VDF-TrFE). (f) Electric field distribution of 10% BT/P(VDF-TrFE). (g) Stress distribution of 20% BT/P(VDF-TrFE). (h) Piezoelectric potential of 20% BT/P(VDF-TrFE). (i) Electric field distribution of 20% BT/P(VDF-TrFE).
